# Psoriasis in Difficult-to-Treat Areas: A Multicentre, Real-World Retrospective Study Analyzing the Impact of Non-Invasive Imaging Techniques (Dermoscopy, Reflectance Confocal Microscopy and Optical Coherence Tomography) to Monitor the Effectiveness of Risankizumab in the Treatment of Plaque Psoriasis of the Legs

**DOI:** 10.3390/clinpract16030046

**Published:** 2026-02-25

**Authors:** Annunziata Dattola, Raimondo Rossi, Giuseppe Rizzuto, Giacomo Caldarola, Eleonora De Luca, Viviana Lora, Domenico Giordano, Severino Persechino, Claudio Bonifati, Diego Orsini, Dario Graceffa, Arianna Zangrilli, Gianluca Pagnanelli, Paola Tribuzi, Annamaria Mazzotta, Gaia Moretta, Adriana Micheli, Alessia Provini, Salvatore Zanframundo, Vincenzo Panasiti, Giovanni Pellacani, Concetta Potenza, Antonio Giovanni Richetta, Nicoletta Bernardini

**Affiliations:** 1 Dermatology Unit, Department of Medical and Cardiovascular Sciences, University of “La Sapienza”, 00161 Rome, Italy; annunziata.dattola@uniroma1.it (A.D.); raimondo.rossi@uniroma1.it (R.R.); pellacani.giovanni@uniroma1.it (G.P.); antonio.richetta@uniroma1.it (A.G.R.); 2 Unità Operativa Complessa di Dermatologia, Dipartimento di Scienze Mediche e Chirurgiche, Fondazione Policlinico Universitario A. Gemelli—IRCCS, 00168 Rome, Italy; giacomo.caldarola@unicatt.it (G.C.); deluca.eleonora94@gmail.com (E.D.L.); 3 Dermatology Unit, Dipartimento di Medicina e Chirurgia Traslazionale, Università Cattolica del Sacro Cuore, 00168 Rome, Italy; 4 Clinical Dermatology Unit, San Gallicano Dermatological Institute (IRCCS), 00144 Rome, Italy; viviana.lora@ifo.it (V.L.); claudio.bonifati@ifo.it (C.B.); dario.graceffa@ifo.it (D.G.); 5 Dermatology Unit, NESMOS Departement, Sant’Andrea Hospital, University of Rome Sapienza, 00189 Rome, Italy; domenico.giordano1989@gmail.com (D.G.); severino.persechino@uniroma1.it (S.P.); 6 Departmental Faculty of Medicine, UniCamillus—“Saint Camillus International University of Health and Medical Sciences”, 00131 Rome, Italy; diego.orsini@unicamillus.org; 7 Unità Operativa Semplice Dipartimentale di Dermatologia, Dipartimento di Scienze Mediche, Azienda Ospedaliera Universitaria Policlinico Tor Vergata, 00133 Rome, Italy; arianna.zangrilli@libero.it; 8 Department of Dermatology, IDI-IRCCS, 00167 Roma, Italy; g.pagnanelli@idi.it (G.P.); gaia.mor@hotmail.it (G.M.); 9 Unità Operativa Semplice Dipartimentale Ospedale Belcolle ASL Viterbo, 01100 Viterbo, Italy; paola.tribuzi@asl.vt.it; 10 Azienda OspedalieraSan Camillo Forlanini of Rome Cir.ne Gianicolense, 00152 Rome, Italy; amazzotta@scamilloforlanini.rm.it; 11 Unità di Dermatologia, Ospedale Nuovo Regina Margherita, 00153 Rome, Italy; micheliadriana@libero.it; 12 Azienda Sanitaria Locale Roma 2, Ospedale S. Pertini, 00157 Rome, Italy; alessiaprovini@yahoo.it; 13 Department of Medicine and Surgery, Università Campus Bio-Medico di Roma, 00128 Rome, Italy; s.zanframundo@gmail.com (S.Z.); v.panasiti@unicampus.it (V.P.); 14 Dermatology Unit “Daniele Innocenzi”, Department of Medical-Surgical Sciences and Biotechnologies, La Sapienza University of Rome, Polo Pontino, 04100 Latina, Italy; concetta.potenza@uniroma1.it (C.P.); nicoletta.bernardini@libero.it (N.B.)

**Keywords:** psoriasis, difficult-to-treat areas, risankizumab, dermoscopy, reflectance confocal microscopy, optical coherence tomography

## Abstract

Objectives: To evaluate the impact of non-invasive imaging techniques such as dermoscopy, reflectance confocal microscopy (RCM) and optical coherence tomography (OCT) to monitor the efficacy of risankizumab on plaque psoriasis of the legs by analyzing morpho-histological changes. Materials and Methods: Multicentre, real-world retrospective study involving 37 adults with moderate-to-severe plaque psoriasis. Assessments performed during routine visits at baseline, Week 4 and Week 12 included clinical response, dermoscopy, RCM and OCT. Results: Thirty-seven patients were included (mean age 52.1 years; 54% male; mean BMI 27.0 kg/m^2^). Dermoscopy showed progressive vascular normalization: at Week 12, 94.29% of lesions had minimal or no vascular pattern. White and yellow scales decreased significantly. On RCM, dilated vessels, inflammatory infiltrate, and papillomatosis progressively normalized. OCT showed reduction in epidermal and stratum corneum thickness and a decline in vascular intensity at multiple depths. Baseline haemorrhagic dots predicted early complete response: 44.8% of lesions with dots achieved complete clearance at Week 4 versus 0% without. Conclusions: Risankizumab induced rapid, significant regression of psoriatic changes, normalizing vascular patterns and skin architecture and reducing epidermal thickness. Findings support its efficacy and rapid onset of action in difficult-to-treat areas and highlight the value of non-invasive imaging for monitoring.

## 1. Introduction

### 1.1. Background

Psoriasis is a chronic immune-mediated inflammatory disease affecting approximately 1–5% of the Western European population [1]. It manifests with well-demarcated, erythematous plaques covered with silvery scales, typically located on the elbows, knees, scalp, and trunk [2]. In addition to skin manifestations, psoriasis is associated with several systemic comorbidities, including psoriatic arthritis, cardiovascular disease, metabolic syndrome, obesity, and psychological disorders, which increase morbidity and significantly reduce quality of life [1,3].

Genetic, immunological, and environmental factors are involved in the pathogenesis of psoriasis, which is therefore complex and multifactorial. A central role is played by the activation of the immune system, particularly the IL-23/Th17 axis, which leads to dysregulated keratinocyte proliferation and the development of a chronic inflammatory response [1,4]. In recent years, advances in understanding the pathogenic mechanisms have led to the development of targeted biological therapies, such as IL-23 inhibitors, which have revolutionized the treatment of moderate-severe psoriasis, offering effective disease control and significant improvement in patients’ quality of life [4].

### 1.2. Psoriasis in Difficult-to-Treat Areas

Before the advent of biologics, the scalp, face, nails, palmoplantar surfaces, genital areas and skin folds were defined as difficult to treat areas [5,6,7,8,9,10,11]. The reason was related to adverse effects and poor patient compliance in the correct application of topical products in areas such as the face, genitals, skin folds or palmoplantar regions [10]. Notably, in a multicenter prospective cohort, calcipotriol/betamethasone foam achieved high satisfaction (TSQM-9) and meaningful PASI reduction at Week 4; nonetheless, adherence and site-specific obstacles can persist in sensitive or recalcitrant locations [12]. Recent evidence suggests that the lower limbs, particularly the pretibial area, should also be considered among difficult-to-treat areas, due to their resistance to treatment and significant impact on patients’ quality of life [6,7,8]. Resistance to traditional treatments as well as biological drugs in the pretibial area is thought to reflect local factors such as venous stasis, gravity, and hydrostatic pressure, which together favour the Koebner phenomenon and sustained inflammation [13]. Furthermore, the histopathology of psoriasis on the legs may differ from classic psoriasis, lacking some hallmark features and sharing histological criteria with stasis dermatitis [14]. Some evidence attributed tissue-resident memory T cells (TRM), a subtype of non-recirculating T cells, an important role in the recurrence of lesions in specific skin sites, acting as an “inflammatory archive” ready to reactivate in the event of new stimuli [15,16]. Resident T lymphocytes express specific markers such as CD103, CCR6 and, most importantly, the IL-23 receptor (IL-23R) [16]. A further study by Matos et al. demonstrated the existence of oligoclonal T lymphocytes in both active and clinically healed psoriatic lesions that expressed TCRβ and TCRα sequences unique to psoriasis and produced IL-17, very similar to TRM [17]. Considering these insights, therapies that neutralize IL-23 may be particularly effective for psoriatic lesions that persistently relapse in these anatomical sites.

Although some studies have assessed the clinical efficacy of anti-IL-23 and anti-IL-17 biologics in difficult-to-treat areas [5,6,7,8,9,18,19] few have examined the accompanying pathophysiological changes in depth. A clearer understanding of these local mechanisms and their response to targeted therapy therefore remains an unmet need.

### 1.3. Non-Invasive High-Resolution Imaging Unmet Needs

The assessment of psoriasis severity and monitoring of treatment response are traditionally based on clinician-reported, semi-quantitative clinical scores, such as the Psoriasis Area and Severity Index (PASI), and on histological examination via skin biopsy [20]. However, these methodologies have some limitations. Clinical scoring systems can be affected by interobserver variability and miss subclinical or early treatment-related changes. Biopsy, while providing detailed information at the cellular and tissue level, is an invasive procedure, not frequently repeatable, and unsuitable for longitudinal monitoring.

Consequently, a significant unmet need remains for non-invasive, objective, and quantitative imaging methods capable of assessing disease severity and monitoring treatment response serially and accurately. Techniques such as dermoscopy, reflectance confocal microscopy (RCM), and optical coherence tomography (OCT) offer the ability to visualize the skin at different depths and resolutions, providing detailed information on skin histology and microcirculation in vivo [21,22,23]. Ultrasound sonoelastography of the hypodermal adipose tissue has also been explored as a complementary modality that quantifies early treatment response via strain-ratio changes in psoriatic plaques [24]. However, robust, prospectively validated imaging biomarkers that translate these qualitative data into valuable endpoints remain scarce, underlining a key unmet need in the management of psoriasis.

Dermoscopy has demonstrated particular utility in the evaluation of psoriasis, allowing for example the identification of the dotted vascular pattern as specific for psoriatic lesions [25]. An important contribution to the role of dermoscopy in psoriasis evaluation and follow-up was made by Lallas et al. In his 2016 study, 75 target lesions in patients who were enrolled for the start of a biological therapy were prospectively monitored. Dermoscopy disclosed key microvascular changes: the progressive disappearance of dotted vessels correlated with plaque clearance and clinical response; the presence of haemorrhagic dots boosted the chances of reaching partial or complete clearance at 4–8 weeks by twenty-fold [26].

RCM was proven by Ardigò et al. to have a >90% concordance with histology for parakeratosis, papillomatosis and dilated papillary vessels [27]. This study put the base for a multicentre adalimumab follow-up in 48 patients, which demonstrated that RCM detects epidermal/dermal inflammatory regression and early repigmentation of dermal papillae as early as Week 4; these changes anticipated PASI improvement and proved predictive of therapeutic response [28]. Complementing these data, Agozzino et al. reviewed confocal follow-up studies and underscored the high reproducibility of RCM metrics for epidermal turnover, vascular calibre and inflammatory cell density while noting that the persistence of large, tortuous capillaries at baseline identifies potential non-responders to systemic therapy [29].

Regarding OCT, Felice et al. demonstrated in a 26-patient cohort on secukinumab that a ≥25% fall in lesional epidermal thickness at Week 4 anticipated earlier PASI-50 attainment, proving OCT can detect sub-clinical improvement before clinical scores change [30]. Complementing this, the systematic review by Guida et al. confirmed that OCT faithfully reproduces the psoriatic hallmarks of hyperkeratosis, serrated dermo-epidermal junction and dilated papillary vessels and correlates them with histology across inflammatory dermatoses [22].

Given the unmet need for objective quantitative lesion-level monitoring in difficult-to-treat anatomical sites and the role of TRM and IL-23 in persistence of psoriatic plaques located on the lower leg, we selected risankizumab as the therapeutic model to evaluate multimodal imaging changes in an index plaque.

### 1.4. Study Endpoints

In this context, this multicentre real-world retrospective study aims to evaluate the effectiveness of risankizumab, a selective IL-23 inhibitor, in the treatment of psoriasis, with a particular focus on lower leg lesions. The primary objective is to analyze the morphological and histological changes induced by risankizumab treatment using high-resolution, non-invasive imaging techniques such as dermoscopy, RCM, and OCT. The secondary endpoint is to correlate these changes with clinical response and evaluate the potential existence of a parameter that could be used as an objective biomarker for predicting therapeutic response and monitoring therapeutic efficacy in a real-world setting.

## 2. Materials and Methods

This multicentre, real-world retrospective study evaluated the morphological and histological changes induced by risankizumab in patients with moderate-to-severe plaque psoriasis, focusing on plaques in the pretibial area (Table S1). Clinical and dermoscopic, RCM and OCT data were retrospectively retrieved from routine visits and imaging archives at baseline (T0), Week 4 (T4) and Week 12 (T12), when available; it also explored the potential existence of a parameter that could be used as an objective biomarker for predicting therapeutic response and monitoring therapeutic efficacy. During routine visits, an index lesion was chosen at baseline as a clinically active plaque located on the pretibial/lower-leg area, and the same plaque was reassessed at each follow-up visit. At each visit, lesional skin was examined in polarized light with a digital dermoscopy system (Vidix 4.0, Canfield Scientific, Parsippany, NJ, USA). A total of 37 patients underwent dermoscopy, a total of 18 patients underwent RCM and a total of 14 patients underwent OCT. OCT was performed using the VivoSight^®^ OCT scanner (Michelson Diagnostics, Maidstone, UK); no keratolytic pre-treatment was used prior to OCT imaging. RCM acquisitions were obtained with the VivaScope 1500 system (Mavig GmbH, Munich, Germany). For each index lesion, clinical response was assessed at Week 4 and Week 12 and categorized as non-responding (NR; no appreciable change or worsening), partially responding (PR; visible improvement without full clearance), or completely remitted (CR; full clinical clearance) based on clinical examination (erythema, scaling, and induration). Clinical response was evaluated independently from imaging. All clinical, dermoscopic, RCM and OCT images were independently reviewed by two experienced dermatologists: RR, GR.

The analysis focused on key dermoscopic parameters, including vascular patterns (regular vessels, dotted vessels, glomerular vessels), scaling (white and yellow scale), the presence of hemorrhagic dots, as well as RCM and OCT parameters. Each parameter was qualitatively scored as present or absent at each timepoint.

To assess changes over time, descriptive statistics were initially performed. Paired comparisons between T0 and T4/T12 were conducted using McNemar’s test for each dermoscopic feature, with statistical significance set at α = 0.05. In addition, a one-sample binomial test was used in selected analyses to test whether the proportion of patients showing change differed significantly from 50%, among those who had the feature at baseline. Graphical visualizations of dermoscopic transitions were constructed using transition diagrams, illustrating the temporal evolution of features between T0, T4 and T12. Bivariate associations between dermoscopic features and clinical response were assessed using chi-square or Fisher’s exact tests, as appropriate. All statistical analyses were performed using SAS software (version 9.4).

## 3. Results

A total of 37 patients were included in this study. The mean age was 52.1 years (SD = 13.0), and the gender distribution was balanced (54.05% males; Table 1). Most patients were overweight or obese, with a mean BMI of 27.0 kg/m^2^ and 70.3% presenting a BMI ≥ 25.

At Week 4, 33/37 (89.2%) lesions were evaluable (4 missed visits): among evaluable lesions, 31/33 (93.9%) achieved at least a response (PR 54.5%, CR 39.4%), while 2/33 (6.1%) showed no response. At Week 12, 36/37 (97.3%) lesions were evaluable (1 missed visit); all evaluable lesions (36/36, 100%) achieved response with complete clearance in 32/36 (88.9%) and partial response in 4/36 (11.1%) lesions.

### 3.1. Dermoscopic Parameters

The following dermoscopic parameters were evaluated at baseline, Week 4, and Week 12 in all 37 patients: vessel distribution, dotted vessels, glomerular vessels, white scale, yellow scale, and haemorrhagic dots. For each dermoscopic parameter, patients with abnormal findings at baseline were identified and grouped; a responder was defined as a patient in whom the feature regressed to a non-pathological pattern (e.g., “none” or “minimal” for vessels, “absent” for scales and dots). For each subgroup, one-sample exact binomial tests were used to evaluate whether the proportion of responders differed significantly from a 50% reference proportion, with statistical significance set at α = 0.05. These analyses were performed independently for Week 4 and Week 12. All statistical analyses were carried out using SAS software (version 9.4).

**Figure 1:**
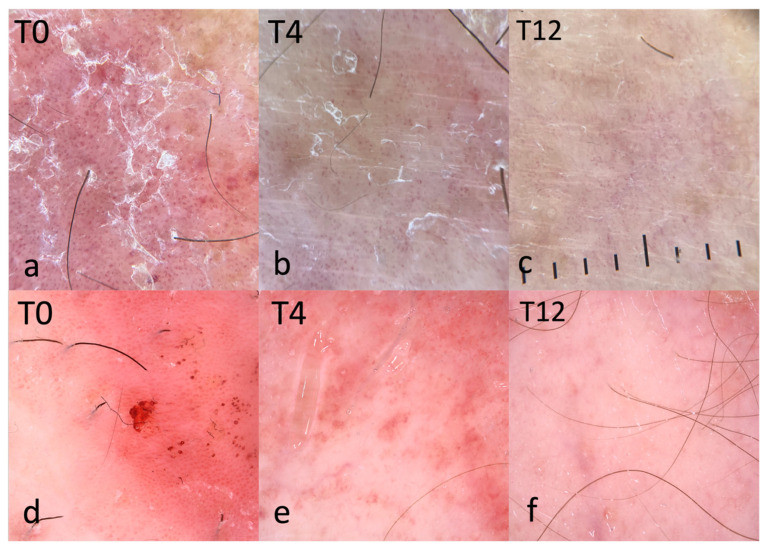
Dermoscopy of a pretibial psoriatic lesion at baseline (**a**,**d**), Week 4 (**b**,**e**), Week 12 (**c**,**f**). Index lesion (**a**–**c**). (**a**) T0: Regularly distributed dotted vessels on an erythematous background with white scales. (**b**) T4: Marked reduction in erythema and scaling, attenuation of dotted vessels with a more clustered distribution. (**c**) T12: near-clearance with sparse residual dotted vessels; erythema and scaling absent. (**d**–**f**) Different patient (representative case with hemorrhagic/purpuric dots). (**d**) T0: hemorrhagic dots associated with uniform dotted vessels. (**e**) T4: early regression of both the vascular pattern and hemorrhagic dots. (**f**) T12: no discernible vascular pattern and no visible scaling. Note: Panels (**d**–**f**) depict a different patient from (**a**–**c**). Polarized dermoscopy. T0, baseline; T4, Week 4; T12, Week 12.

**Figure 2:**
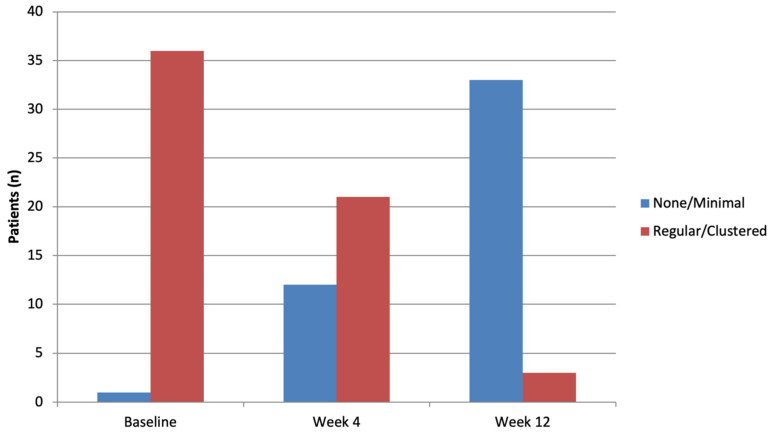
Dermoscopic vessel distribution over time (none/minimal versus regular/clustered). A clear shift toward none/minimal is observed over time; differences versus T0 are significant at both follow-ups (McNemar, T4 *p* = 0.002; T12 *p* < 0.0001).

A progressive improvement in dermoscopic vascular morphology was observed (Figure 1). At baseline, vessel distribution (Table 2 and Figure 2; Table S2 and Figure S1) was predominantly regular or clustered, with only one patient showing a minimal pattern. By Week 4, a clear shift was evident with over a third of lesions showing a minimal pattern (12/33, 36.4%, *p* = 0.0022). At Week 12, 26/36 (72.2%) of the lesions showed no vascular pattern, while only one lesion remained clustered (*p* < 0.0001). Among the 36 patients who presented a regular or clustered vessel distribution at baseline, 12/36 (37.5%) showed regression to a minimal or absent pattern at Week 4 (*p* = 0.1076). By Week 12, this proportion increased markedly, with 33/36 (94.3%) patients showing resolution of the vascular pattern (*p* < 0.0001).

A similar trend was noted for dotted vessels (Figure S2). Initially, 26/37 (70.3%) lesions displayed a “uniform” pattern. While no significant variation was seen at Week 4, by Week 12, 28/26 (77.8%) lesions showed complete absence of dotted vessels (*p* < 0.0001), suggesting a strong treatment-related regression. In the subgroup of patients with uniform or clustered dotted vessels at baseline (n = 27), only 1/27 (4.2%) showed regression to an absent or unspecific pattern at Week 4. However, by Week 12, 21/27 (77.8%) patients had regressed (*p* = 0.0029).

The distribution of glomerular vessels (Figure S3) remained unchanged between baseline and Week 4 (*p* = 0.6547). However, at Week 12, 30/36 (83.3%) lesions showed no glomerular vessels, with a marked reduction in “uniform” and “clustered” patterns (*p* < 0.0001). Similarly, of the 21 patients with uniform or clustered glomerular vessels at baseline, 18/21 (85.7%) no longer exhibited these patterns at Week 12 (*p* = 0.0007). This association was not yet significant at Week 4 (8/21, 36.4%, *p* = 0.1431).

At baseline, hemorrhagic dots were present in 29/37 lesions (78.4%; Table S3 and Figure S4). Prevalence fell sharply to 8/33 (24.2%) at Week 4 (*p* < 0.0001 vs. baseline) and to 0/36 (0%) at Week 12 (all evaluable lesions cleared). Among lesions positive at baseline, 20/29 (74.1%) showed disappearance already at Week 4 (*p* = 0.0095 vs. 50%), rising to 29/29 (100%) at Week 12 (*p* < 0.0001; Figure 1d,e). Clinical cross-tabulations showed that baseline presence of hemorrhagic dots was associated with a higher rate of complete response at Week 4 (44.8% with dots vs. 0% without; *p* = 0.0187), while the difference was not significant at Week 12 (89.7% vs. 75.0%; *p* = 0.2830; Table 3).

**Figure 3:**
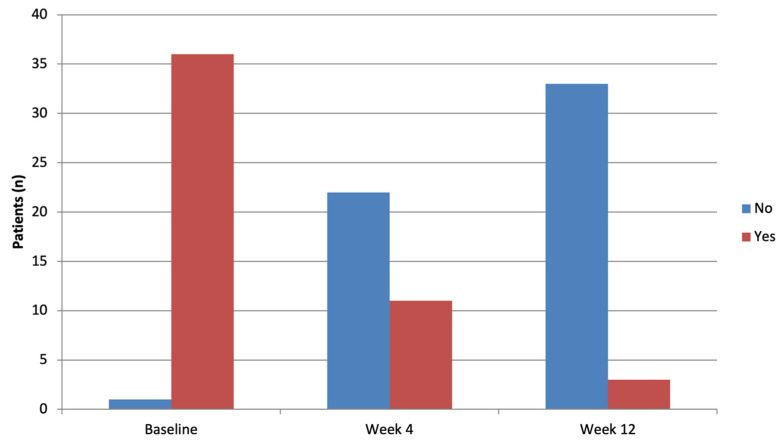
Bar chart showing the prevalence of white scale at baseline (T0), Week 4 (T4), and Week 12 (T12). The reduction versus T0 is significant at both follow-ups (McNemar test *p* < 0.0001).

Scaling also improved over time. White scale (Figure 1 and Figure 3; see also Tables S4 and S5), observed in nearly all patients at baseline (36/37, 97.3%), decreased rapidly to 11/33 (33.3%) at Week 4 (*p* < 0.0001), and further to 3/36 (8.3%) at Week 12 (*p* < 0.0001). At Week 4, 21/36 (58.33%) of the patients who had white scale at baseline no longer showed white scale, although this did not reach statistical significance. At Week 12, the count rose to 32/36 (88.9%), with a significant association observed (*p* < 0.0001). Yellow scale, initially present in 9/38 (24.3%) of patients, showed a non-significant decrease at Week 4 (3/33, 9.1%, *p* = 0.1572) but was completely absent by Week 12 (0/36, 0%).

### 3.2. RCM Parameters

The following RCM parameters were evaluated at baseline, Week 4, and Week 12 in a subgroup of 18 patients: dilated vessels, inflammatory infiltrate, and papillomatosis.

**Figure 4:**
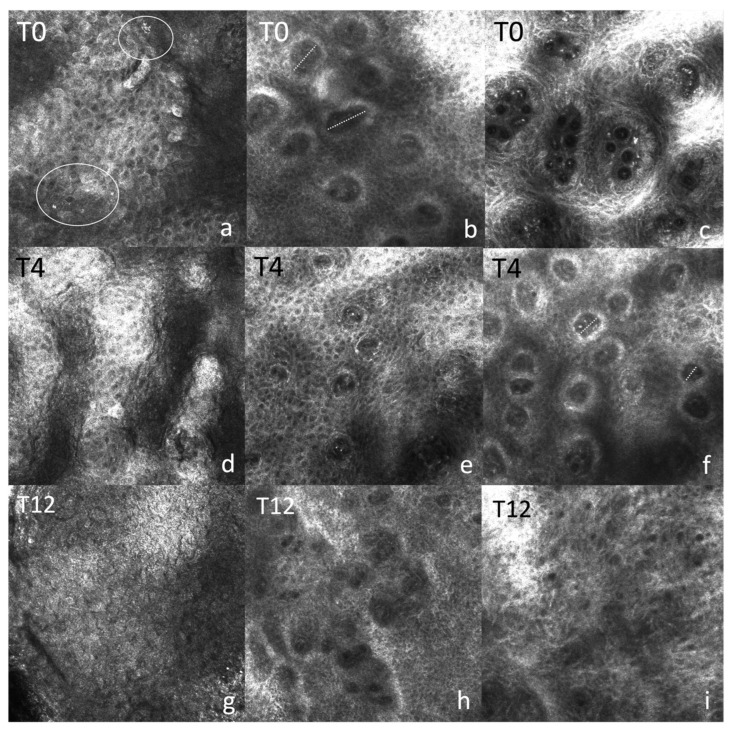
Reflectance confocal microscopy at baseline (**a**–**c**), Week 4 (**d**–**f**), and Week 12 (**g**–**i**). (**a**–**c**) T0: epidermal papillomatosis with enlarged dermal papillae; dilated vessels (dashed lines); inflammatory infiltrate (white circle). (**d**–**f**) T4: reduction in vessel calibre and attenuation of papillomatosis; inflammatory infiltrate no longer detectable. (**g**–**i**) T12: architectural and vascular normalization, with absence of papillomatosis and inflammatory cells. RCM, reflectance confocal microscopy; T0, baseline; T4, Week 4; T12, Week 12.

A progressive normalization was observed across all parameters (Figure 4). Dilated vessels were prominent at baseline, with most patients showing involvement ≥50% of the imaged area (Table S6 and Figure S5); by Week 12, the vast majority (11/13, 84.6%) showed <25% involvement. Exact binomial test confirmed a progressive improvement over time, with the proportion of patients exhibiting <50% vascular area increasing from 66.7% at Week 4 to 87.5% at Week 12 (*p* = 0.0351).

**Figure 5:**
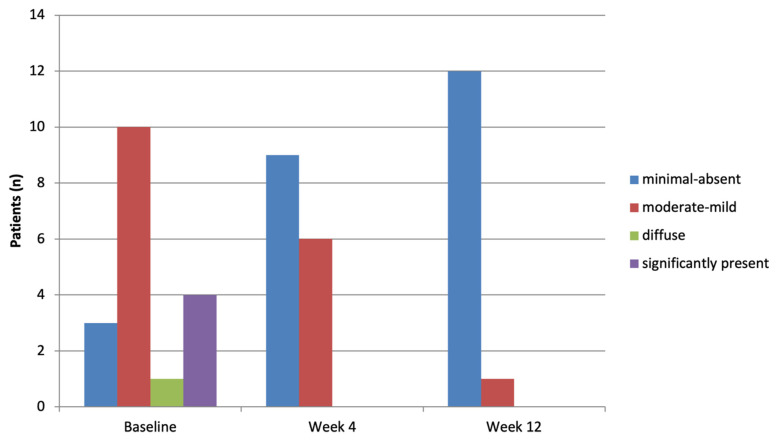
RCM inflammatory infiltrate over time. RCM: reflectance confocal microscopy.

Inflammatory infiltrate (Figure 5; see also Table S7) was also common at baseline but steadily decreased over time, with only minimal signs remaining at the end of treatment, with all patients (15/15, 100%) showing minimal to mild infiltrate by Week 4 and maintaining this pattern at Week 12 (*p* = 0.0625).

Papillomatosis, initially present in 15/18 (83.3%) cases, showed limited improvement at Week 4 but disappeared by Week 12 in 12/13 (92.3%) patients (Table S8 and Figure S6).

### 3.3. OCT Parameters

The following OCT parameters were evaluated at baseline, Week 4, and Week 12 in a subgroup of 14 patients: epidermal thickness, stratum corneum thickness, and vascular intensity at depths of 150, 300, and 500 µm.

**Figure 6:**
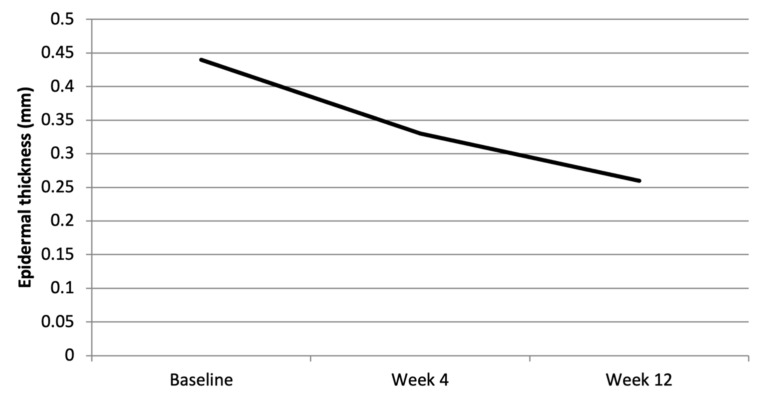
OCT epidermal thickness over time (mean). OCT: optical coherence tomography.

**Figure 7:**
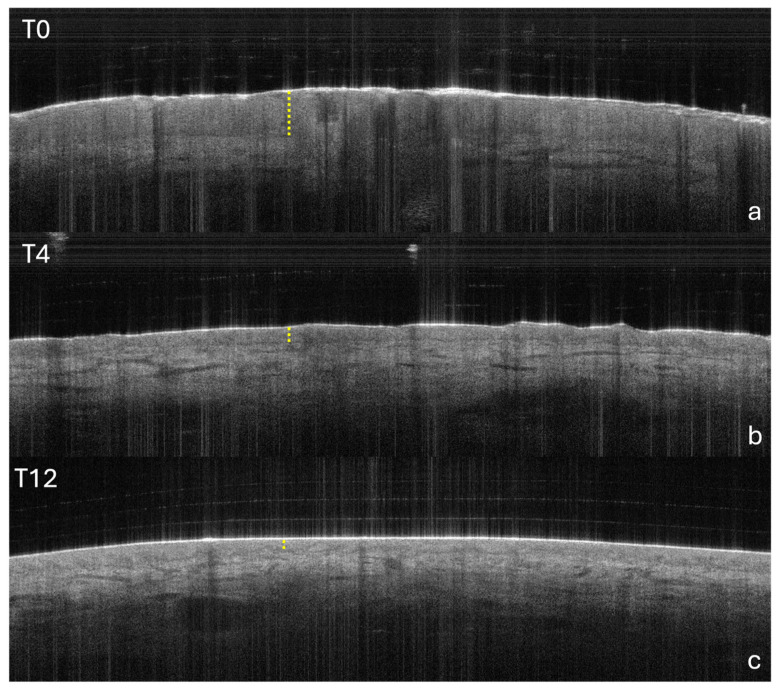
Optical coherence tomography (OCT) at baseline (**a**), Week 4 (**b**), and Week 12 (**c**). A progressive reduction in epidermal thickness (dashed yellow lines) is evident from T0 to T12, consistent with treatment response. OCT, optical coherence tomography; T0, baseline; T4, Week 4; and T12, Week 12.

Epidermal thickness (Figure 6 and Figure 7, see also Table S9) progressively decreased over time, from a mean of 440 µm at baseline to 260 µm at Week 12, reflecting consistent epidermal remodelling. A similar trend was observed for the stratum corneum, which showed an initial mean thickness of 70 µm that dropped to 30 µm by Week 12 (Table S10 and Figure S7). Among the patients with a baseline epidermal thickness greater or equal to 400 µm, 7/9 (77.8%) at Week 4 and 8/9 (88.9%) at Week 12 (*p* = 0.0195) showed a consistent reduction <400 µm, and the same proportions were observed for stratum corneum thickness <60 µm, supporting a parallel normalization in epidermal architecture.

Vascular intensity showed more variability, particularly at 150 µm, where a transient increase was observed at Week 4 before a sharp reduction at Week 12. At deeper levels (300 and 500 µm), vascular signals were initially high (mean values of 11,870 and 13,187, respectively) and declined substantially over time, with Week 12 values reduced to 2256.9 and 7551.7 (Tables S11, S12 and S13; Figures S8, S9 and S10). At each depth threshold (1300; 8500; and 4900 units, respectively), the proportion of patients showing a reduction below threshold increased from ~21.4% at Week 4 to 85.7–92.9% at Week 12, with statistically significant differences (*p*-values ranging from 0.0286 to 0.0009).

## 4. Discussion

### 4.1. Main Results

This multicentre, real-world, retrospective study explored the efficacy of risankizumab, a selective IL-23 inhibitor, adopting an index lesion-targeted design with a particular focus on lower legs/pretibial plaques, using non-invasive imaging techniques. Clinical response was evaluated at the index lesion level (NR/PR/CR) and interpreted alongside imaging findings: 93.9% achieved at least a partial response at Week 4 (CR 39.4%), while at Week 12 complete clearance was observed in 32/36 lesions (88.9%). Our results demonstrate rapid and significant regression of psoriasis-associated morphological and histological changes as early as Week 4, with marked improvement at Week 12. These data support the efficacy of risankizumab even in difficult-to-treat areas and highlight the value of non-invasive imaging for monitoring treatment response.

Dermoscopic analysis revealed a progressive improvement in vascular morphology. At Week 12, 94.29% of lesions showed none or minimal vessel distribution pattern, with a significant decrease in dotted vessels (77.78% complete absence at Week 12) as well as white and yellow scale. These results are consistent with the rapid resolution of inflammation and keratinocyte hyperproliferation induced by risankizumab.

RCM confirmed a progressive normalization of dilated vessels, inflammatory infiltrate, and papillomatosis. In particular, dilated vessels show a clear numerical improvement by Week 4 (from 14/18 to 4/15 patients with involvement > 50%), however, not reaching statistical significance due to the small sample size. At baseline, 15/18 lesions showed some degree of inflammatory infiltrate; by Week 4, the distribution inverted, with 9/15 lesions (60%) already showing minimal or absent inflammatory infiltrate, culminating at T12 with 12/13 showing minimal-to-none RCM signs of inflammation. This stepwise shift demonstrated the rapid clearance of the inflammatory infiltrate already at Week 4 that consolidates over time, despite the progressive drop in evaluable lesions. Papillomatosis showed a significant change at Week 12, dropping from 80% to 7.7%, confirming a delayed but marked epidermal structural normalization. However, only 18 of 37 participants had RCM at baseline, and further losses occurred at Week 4 (n = 15). This greatly reduced statistical power, explaining why large relative improvements did not translate into statistical significance.

OCT showed a progressive reduction in epidermal thickness (from 0.44 mm to 0.26 mm at Week 12) and stratum corneum thickness (from 0.07 mm to 0.03 mm at Week 12), with a decrease in vascular intensity at different depths. These objective data, obtained with non-invasive techniques, corroborate the clinical efficacy of the drug and provide a deeper understanding of the mechanisms of action at tissue level, confirming the usefulness of the integrated dermoscopy-RCM-OCT approach in monitoring the resolution of psoriatic plaques.

### 4.2. The Literature Comparison

Our study reinforces the importance of non-invasive imaging techniques as complementary tools for monitoring treatment response. The literature has already highlighted the value of dermoscopy, OCT and RCM in the diagnosis and follow-up of psoriasis [22,23,25,26,27,28,29]. The standardization of dermoscopic terminology for non-neoplastic dermatoses, as proposed by the international consensus [21], provides a robust framework for the interpretation of our results. Our detailed analysis of changes in vascular patterns and epidermal structures provides further evidence of their potential as objective biomarkers of therapeutic response, partially overcoming the limitations of semi-quantitative clinical scoring systems.

Furthermore, our findings focused on the lesions on the pretibial area are consistent with growing evidence supporting the efficacy of risankizumab in the treatment of moderate-to-severe plaque psoriasis, both in pivotal clinical trials and in real-world settings [5,6,7,9,19,31,32], several of which have demonstrated that risankizumab induces high and durable clinical responses. Our observation of rapid normalization of dermoscopic, RCM, and OCT parameters aligns with data indicating a rapid onset of action of risankizumab, with significant improvements as early as 4 weeks.

### 4.3. Pathophysiological Significance of RCM/OCT Markers

The changes observed by RCM and OCT, such as the reduction in dilated vessels, inflammatory infiltrate, papillomatosis, and normalization of epidermal and stratum corneum thickness, reflect the pathophysiological mechanisms underlying psoriasis and their modulation by risankizumab. Psoriasis is characterized by keratinocyte hyperproliferation, inflammation, and angiogenesis [1,2,3,4]. The decrease in dilated vessels and inflammatory infiltrate observed with RCM indicates a reduction in inflammation and angiogenesis, key processes in the pathogenesis of the disease.

The correlation between RCM features and horizontal histological sections, as demonstrated in previous studies [27,28,29], confirms that our observations accurately reflect the underlying histological changes. The normalization of epidermal and stratum corneum thickness, highlighted by OCT, is a direct sign of the resolution of keratinocyte hyperproliferation and parakeratosis, histopathological hallmarks of psoriasis. Moreover, our findings confirm and reinforce those obtained by Ardigo et al., Agozzino et al. and Felice et al. regarding the effect of biologic therapies on tissue-imaging changes in psoriatic lesions [27,28,29,30].

These non-invasive imaging markers offer an in vivo window into changes at the tissue level, providing valuable information about the biological response to treatment that is not always evident with clinical assessment alone.

### 4.4. Predictive Value of Haemorrhagic Dots

An interesting aspect that emerged from our study is the predictive value of haemorrhagic dots. Haemorrhagic dots were the dermoscopic parameter with the fastest regression kinetics observed in this study. Their prevalence increased from 78.4% at baseline to 24.2% as early as week 4, reaching zero at week 12.

Bivariate analysis showed that the presence of haemorrhagic dots at T0 did not discriminate between patients who would achieve any early clinical response, as all subjects with (29/29) or without (8/8) dots achieved at least a partial response at T4. However, the presence of dots at baseline significantly correlates with the achievement of an early complete response: 44.8% of patients with dots achieved a complete clinical response compared to 0% of those without (χ^2^, *p* = 0.0187).

Further confirmation comes from the responder analysis: among patients initially positive for haemorrhagic dots, 74.1% already showed the absence of the sign at T4 (*p* = 0.0095 versus a hypothetical threshold of 50%), a percentage that reached 100% at T12. These results indicate that the early disappearance of haemorrhagic dots reflects rapid vascular normalization, and the presence of the sign at baseline can be considered an indicator of a complete response. The observed dynamics support the hypothesis of Lallas et al. [26] according to which haemorrhagic dots constitute a dermoscopic biomarker of early response to biologics.

### 4.5. Limitations and Strengths

This study has several strengths. The use of non-invasive, high-resolution imaging techniques (dermoscopy, RCM, OCT) represents an innovative and objective approach to assessing treatment response, providing quantitative and qualitative data on morphological and histological changes in vivo. The focus on lower limbs as difficult-to-treat areas is particularly relevant given the therapeutic challenge these areas represent.

However, this study also has some limitations. The sample size could be increased to further confirm the results. Global PASI was not collected. The retrospective nature of this study precludes causal inference and may be affected by selection bias and incomplete data, particularly for imaging. Importantly, as expected in a real-world multicentre setting, the availability of RCM and OCT and follow-up imaging completion varied across centres, which may affect generalisability and highlights the need for standardized acquisition workflows in future studies. Future research should include randomized, controlled trials with larger sample sizes and long-term follow-up to consolidate these findings.

### 4.6. Clinical Implications and Future Research

The clinical implications of this study are significant. Our results strengthen the efficacy profile of risankizumab, demonstrating its ability to induce rapid and profound resolution of psoriatic lesions, even in traditionally difficult-to-treat areas. The integration of non-invasive imaging techniques into clinical practice could improve treatment response monitoring, allowing for more objective and earlier assessment of efficacy and facilitating personalized treatment decisions. This is particularly important for difficult-to-treat areas, where clinical assessment can be more complex.

Future research should focus on further validating these imaging biomarkers in larger and more diverse patient cohorts. It would be beneficial to explore the correlation between changes observed with imaging and long-term outcomes, such as disease remission and prevention of relapses. Furthermore, comparative studies with other biologic agents could provide a more comprehensive understanding of Risankizumab’s placement in the psoriasis treatment algorithm. Finally, the application of artificial intelligence and machine learning techniques to the analysis of imaging data could further improve the predictive capacity of these tools, opening new perspectives for precision medicine in psoriasis.

### 4.7. Conclusions

This study demonstrated that risankizumab induces rapid and significant improvement of the psoriatic lesions in the lower leg/pretibial area. High-resolution non-invasive imaging techniques, such as dermoscopy, RCM, and OCT, revealed normalization of vascular patterns, reduced inflammation, and reduced epidermal hyperproliferation. These may represent a useful complement for objectively monitoring treatment response. In particular, the presence of haemorrhagic dots suggests a significant early response indicator. Our results strengthen the role of risankizumab as an effective therapeutic option in difficult-to-treat areas affecting the lower leg, particularly pretibial lesions, and support the integration of non-invasive imaging for more precise and personalized management of psoriasis. Future larger studies should validate these imaging biomarkers and assess their reproducibility across centres.

## Figures and Tables

**Table 1:** Demographics and anthropometric characteristics of the study cohort. BMI: body mass index; SD: standard deviation.

Parameter	Value (Overall Set = 37)
Mean age, years ± SD	52.1 ± 13.0
Gender, n (%)	
Male	20 (54.05)
Female	17 (45.95)
Mean weight, kg ± SD	76.5 ± 16.4
BMI	
Mean ± SD	27.0 ± 4.7
≥25, n (%)	26 (70.27)
<25, n (%)	11 (29.73)

**Table 2:** Dermoscopic vessel distribution over time (none/minimal versus regular/clustered).

Visit	Modality	Statistics	Overall Set N = 37	*p*-Value
Baseline		N	37	
		N missing	0	
	None/Minimal	N (%)	1 (2.70)	
	Regular/Clustered	N (%)	36 (97.30)	
Week 4		N	33	
		N missing	4	
	None/Minimal	N (%)	12 (36.36)	0.0022
	Regular/Clustered	N (%)	21 (63.64)	
Week 12		N	36	
		N missing	1	
	None/Minimal	N (%)	33 (91.67)	<0.0001
	Regular/Clustered	N (%)	3 (8.33)	

**Table 3:** Baseline hemorrhagic dots and clinical response at Week 4 and Week 12. CR: completely remitted; NR: non-responding; PR: partially responding; and HD: hemorrhagic dots.

Visit	Modality	Statistics	Baseline HD No	Baseline HD Yes	*p*-Value
			N = 8	N = 29	
Week 4		N	8	29	
		N missing	0	0	
	CR	N (%)	0	13 (44.83)	0.0187
	NR/PR	N (%)	8 (100)	16 (55.17)	
Week 12		N	8	29	
		N missing	0	0	
	CR	N (%)	6 (75.00)	26 (89.66)	0.2830
	NP/NR	N (%)	2 (25.00)	3 (10.34)	

## Data Availability

Data (demographics, clinical variables and dermoscopic, RCM and OCT outputs) and the analysis code are available from the corresponding author upon reasonable request, subject to institutional approvals and data-sharing agreements. Data are not publicly available due to privacy restrictions.

## Supplementary Materials

The following supporting information can be downloaded at: https://www.mdpi.com/article/10.3390/clinpract16030046/s1, Table S1: Frequency of subjects by site. Table S2: Dermoscopic Vessels Distribution over time. Table S3: Dermoscopic Haemorragic Dots distribution over time. Table S4: Dermoscopic White Scale distribution over time. Table S5: Dermoscopic Yellow Scale distribution over time. Table S6: Dilated Vessels distribution on Reflectance Confocal Microscopy over time. Table S7: Inflammatory Infiltrate distribution on Reflectance Confocal Microscopy over time. Table S8: Papillomatosis distribution on Reflectance Confocal Microscopy over time. Table S9: Epidermal Thickness on Optical Coherence Tomography over time. Table S10: Stratum Corneum Thickness on Optical Coherence Tomography over time. Table S11: Vascular intensity at 150 µm depth on Optical Coherence Tomography over time. Table S12: Vascular intensity at 300 µm depth on Optical Coherence Tomography over time. Table S13: Vascular intensity at 500 µm depth on Optical Coherence Tomography over time. Figure S1: Dermoscopic Vessels Distribution over time. Figure S2: Dermoscopic Dotted Vessels Distribution over time. Figure S3: Dermoscopic Glomerular Vessels Distribution over time. Figure S4: Dermoscopic Haemorragic Dots Distribution over time. Figure S5: Dilated Vessels distribution on Reflectance Confocal Microscopy over time. Figure S6: Papillomatosis distribution on Reflectance Confocal Microscopy over time. Figure S7: Stratum Corneum Thickness on Optical Coherence Tomography over time. Figure S8: Vascular intensity at 150 µm depth on Optical Coherence Tomography over time. Figure S9: Vascular intensity at 300 µm depth on Optical Coherence Tomography over time. Figure S10: Vascular intensity at 500 µm depth on Optical Coherence Tomography over time.
