# Supplementary figures and images for: Psoriasis in Difficult-to-Treat Areas: A Multicentre, Real-World Retrospective Study Analyzing the Impact of Non-Invasive Imaging Techniques (Dermoscopy, Reflectance Confocal Microscopy and Optical Coherence Tomography) to Monitor the Effectiveness of Risankizumab in the Treatment of Plaque Psoriasis of the Legs
Source: Clin Pract. 2026 Feb 25;16(3):46. doi: 10.3390/clinpract16030046 (PMC13025591; doi:10.3390/clinpract16030046)

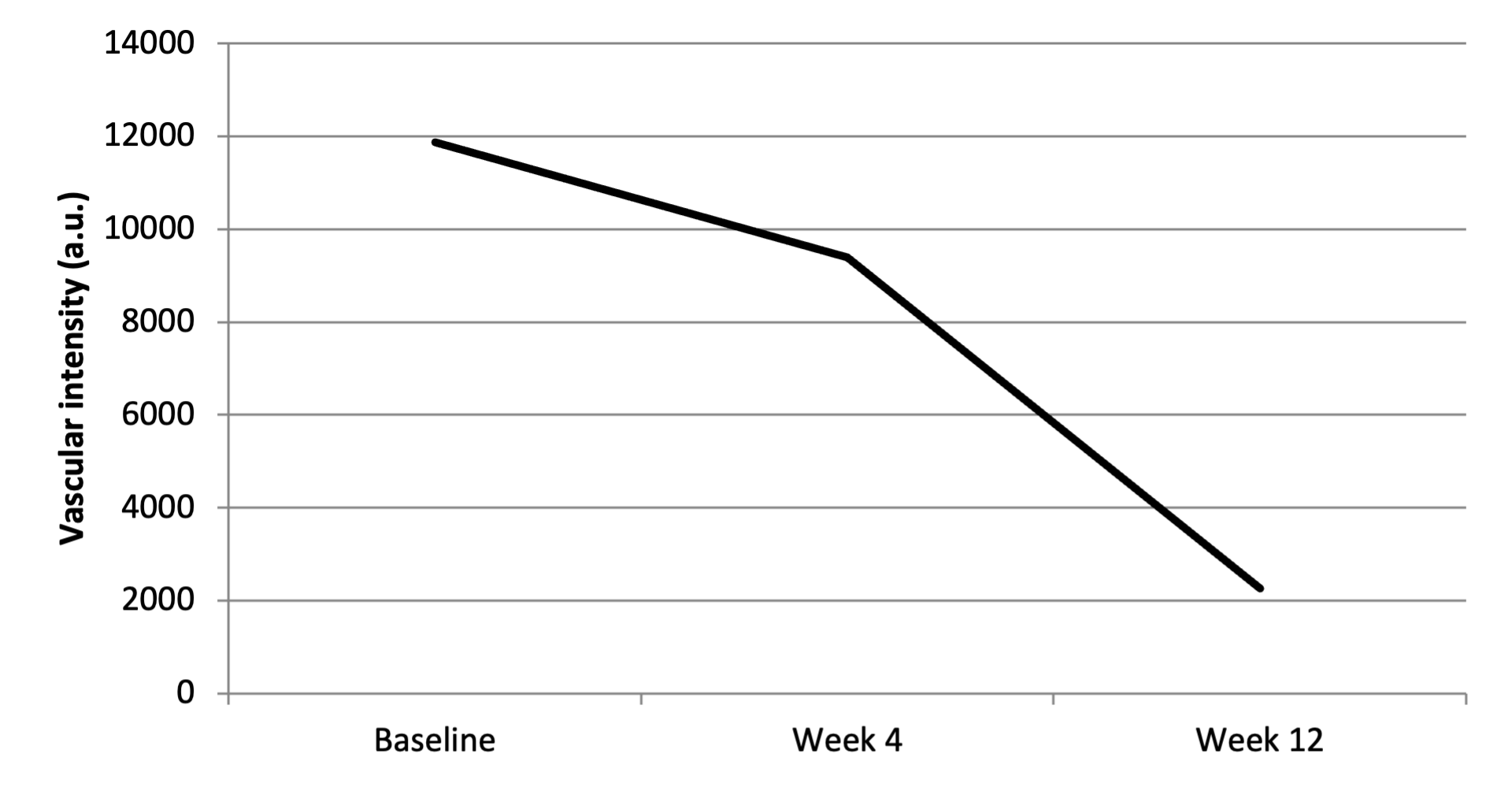

Supplement: Supplementary file 1 [file clinpract-16-00046-s001.zip › Supplementary/FigureS9.png]

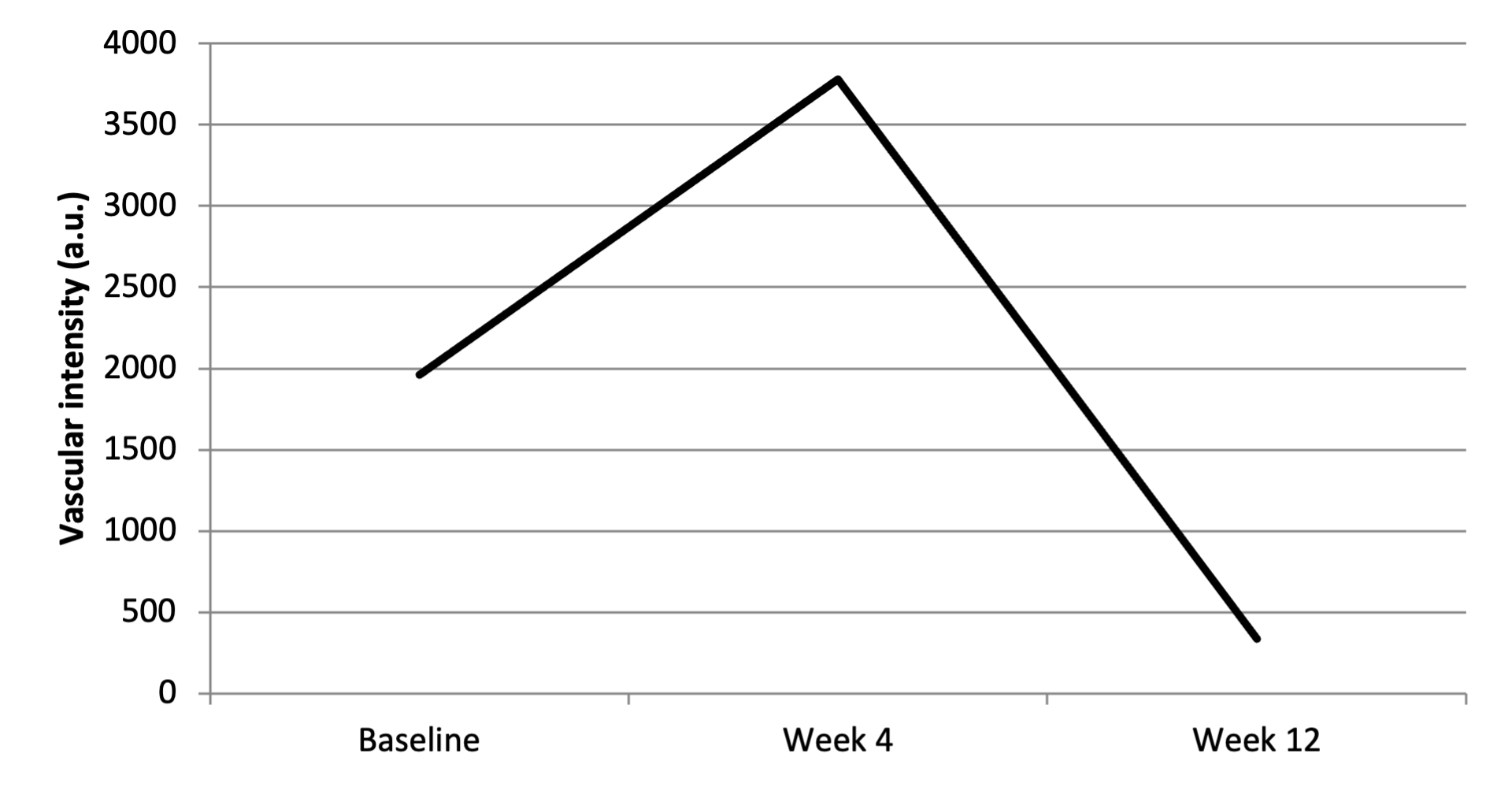

Supplement: Supplementary file 1 [file clinpract-16-00046-s001.zip › Supplementary/FigureS8.png]

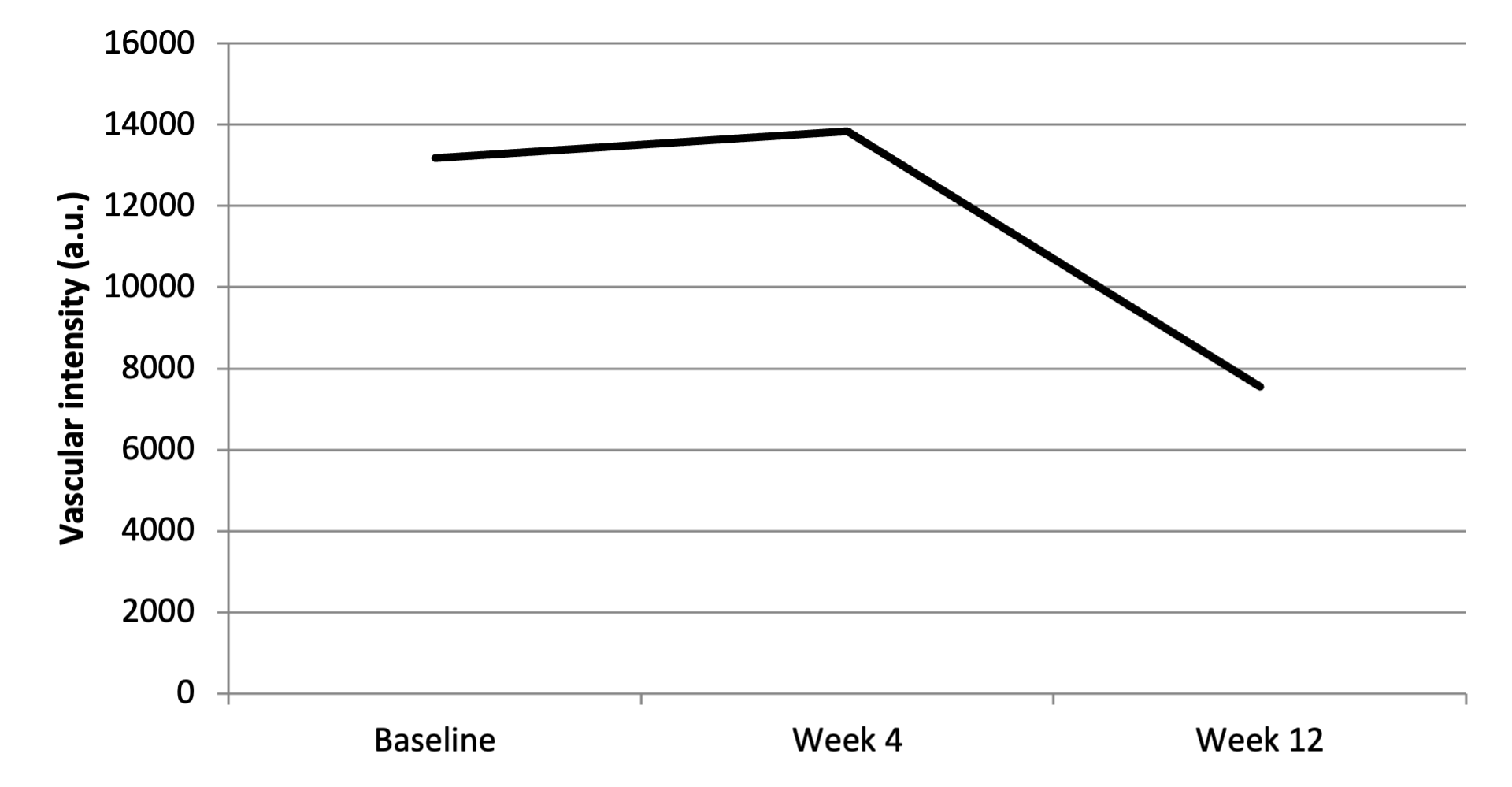

Supplement: Supplementary file 1 [file clinpract-16-00046-s001.zip › Supplementary/FigureS10.png]

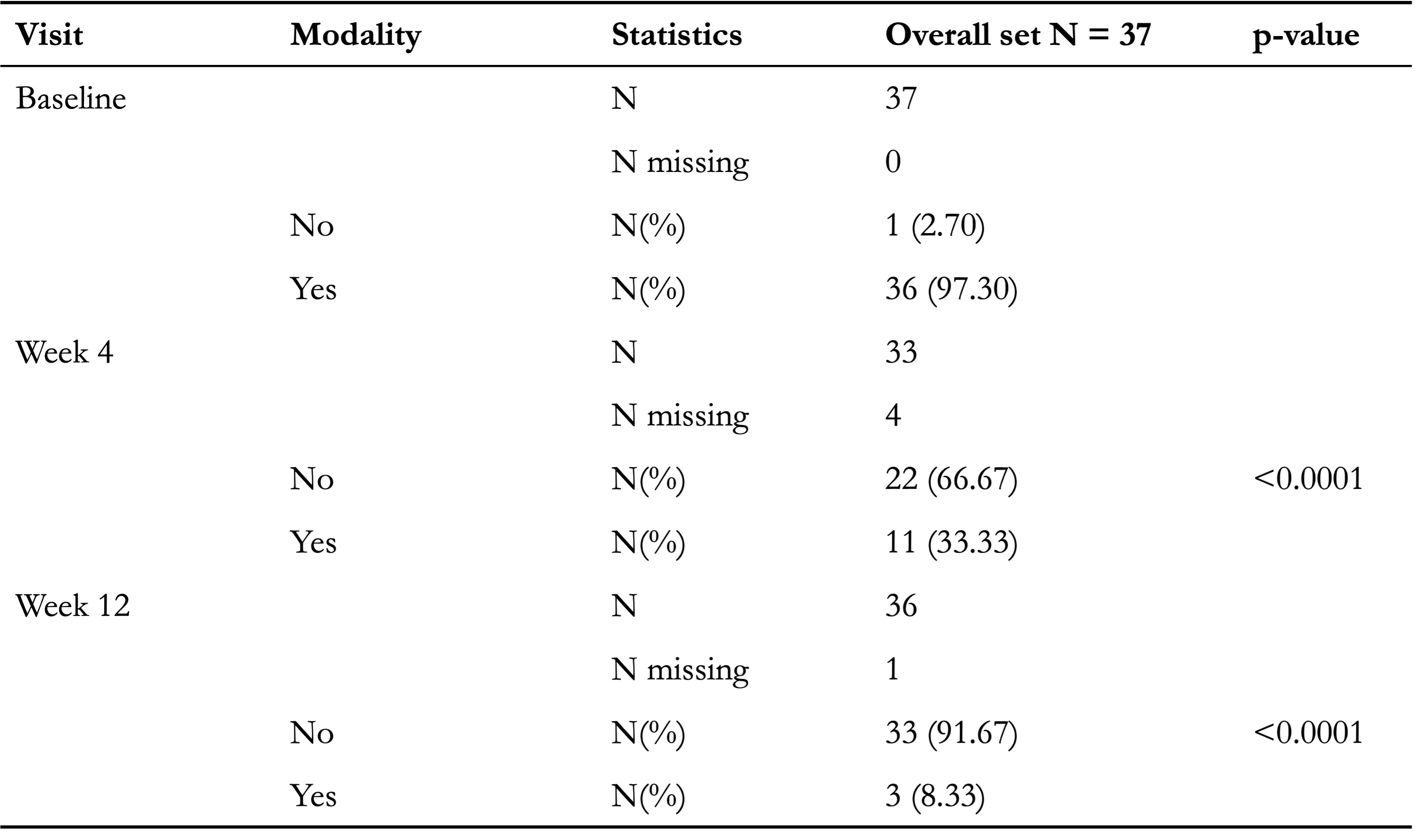

Supplement: Supplementary file 1 [file clinpract-16-00046-s001.zip › Supplementary/TableS4.png]

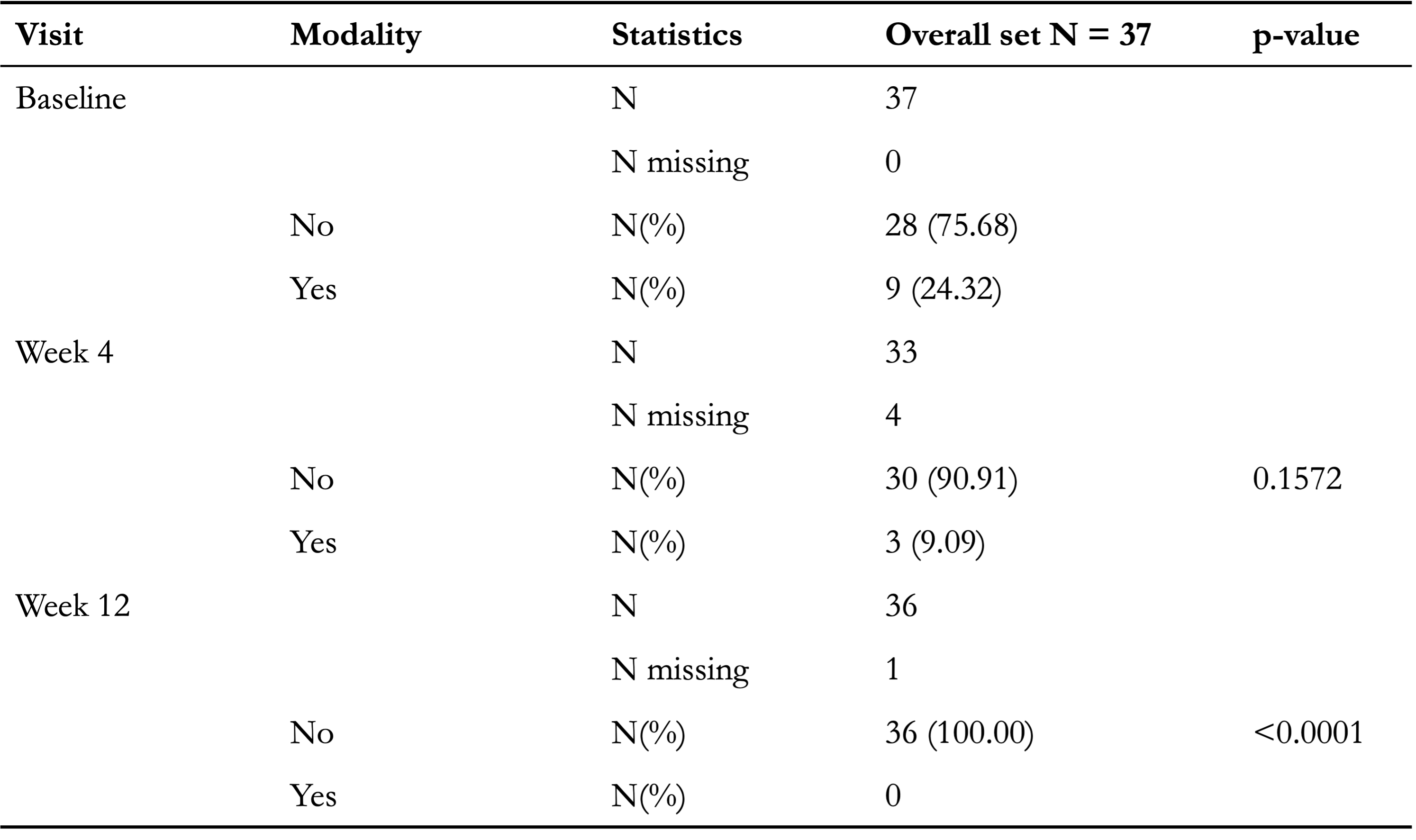

Supplement: Supplementary file 1 [file clinpract-16-00046-s001.zip › Supplementary/TableS5.png]

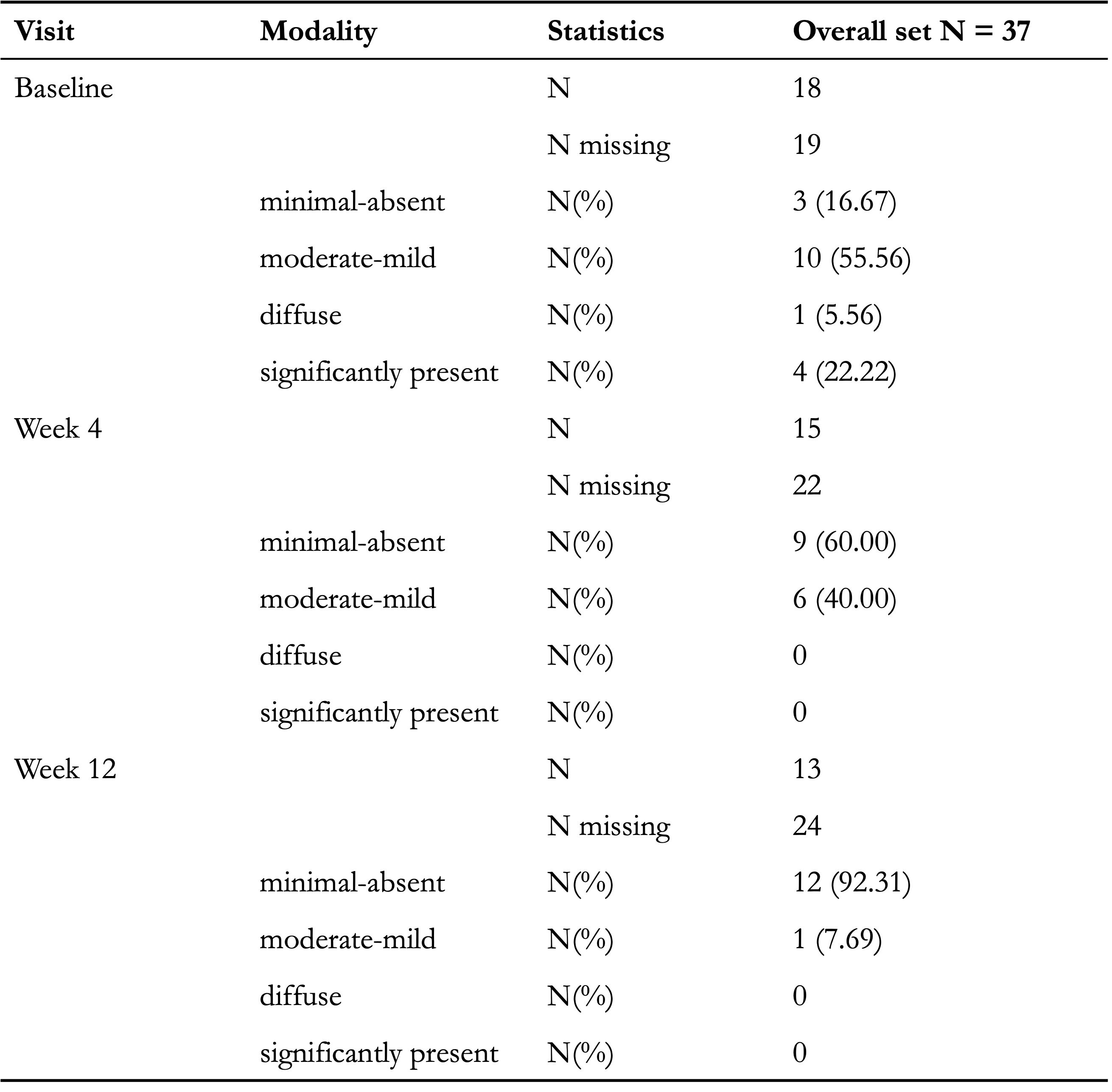

Supplement: Supplementary file 1 [file clinpract-16-00046-s001.zip › Supplementary/TableS7.png]

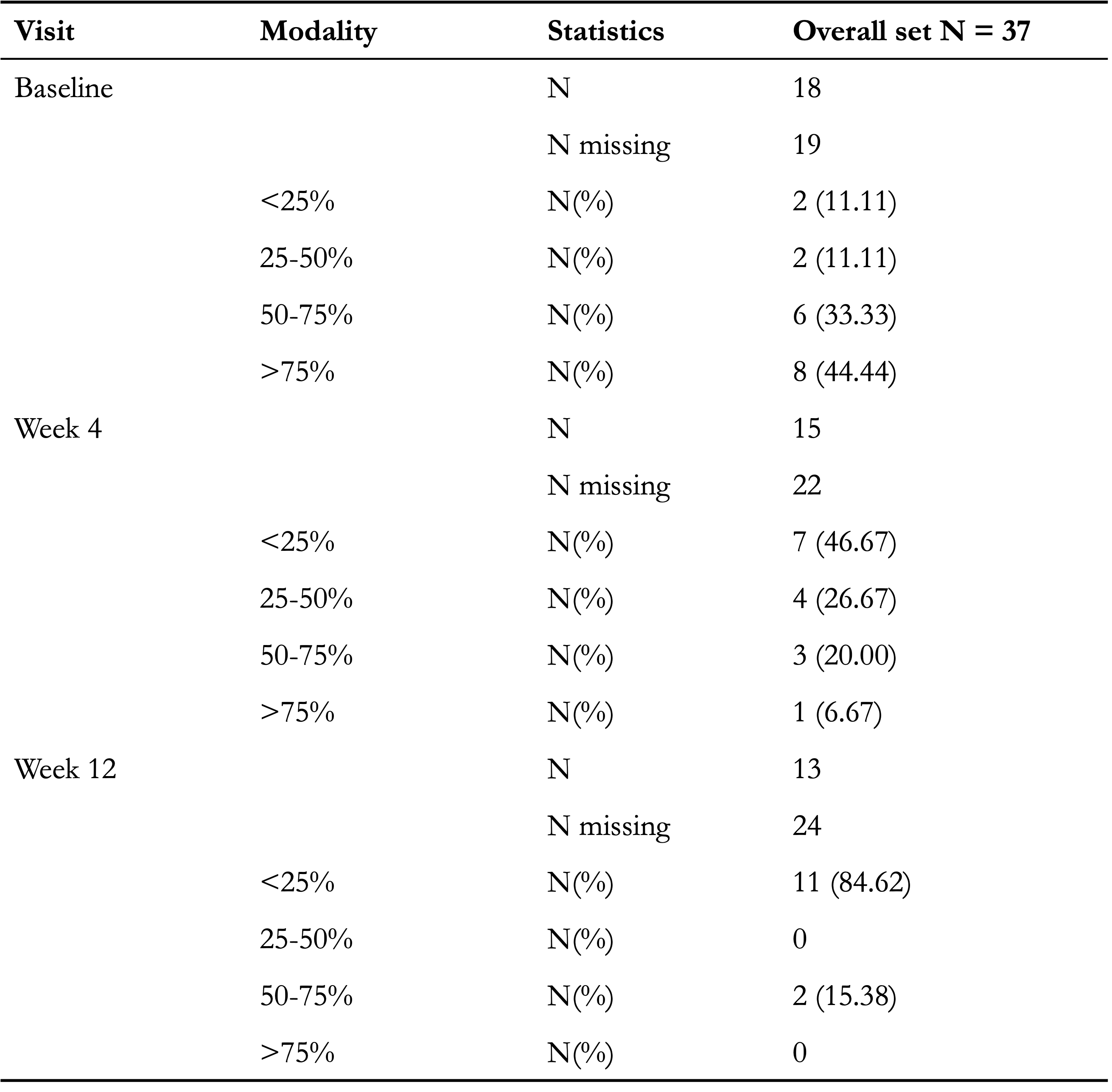

Supplement: Supplementary file 1 [file clinpract-16-00046-s001.zip › Supplementary/TableS6.png]

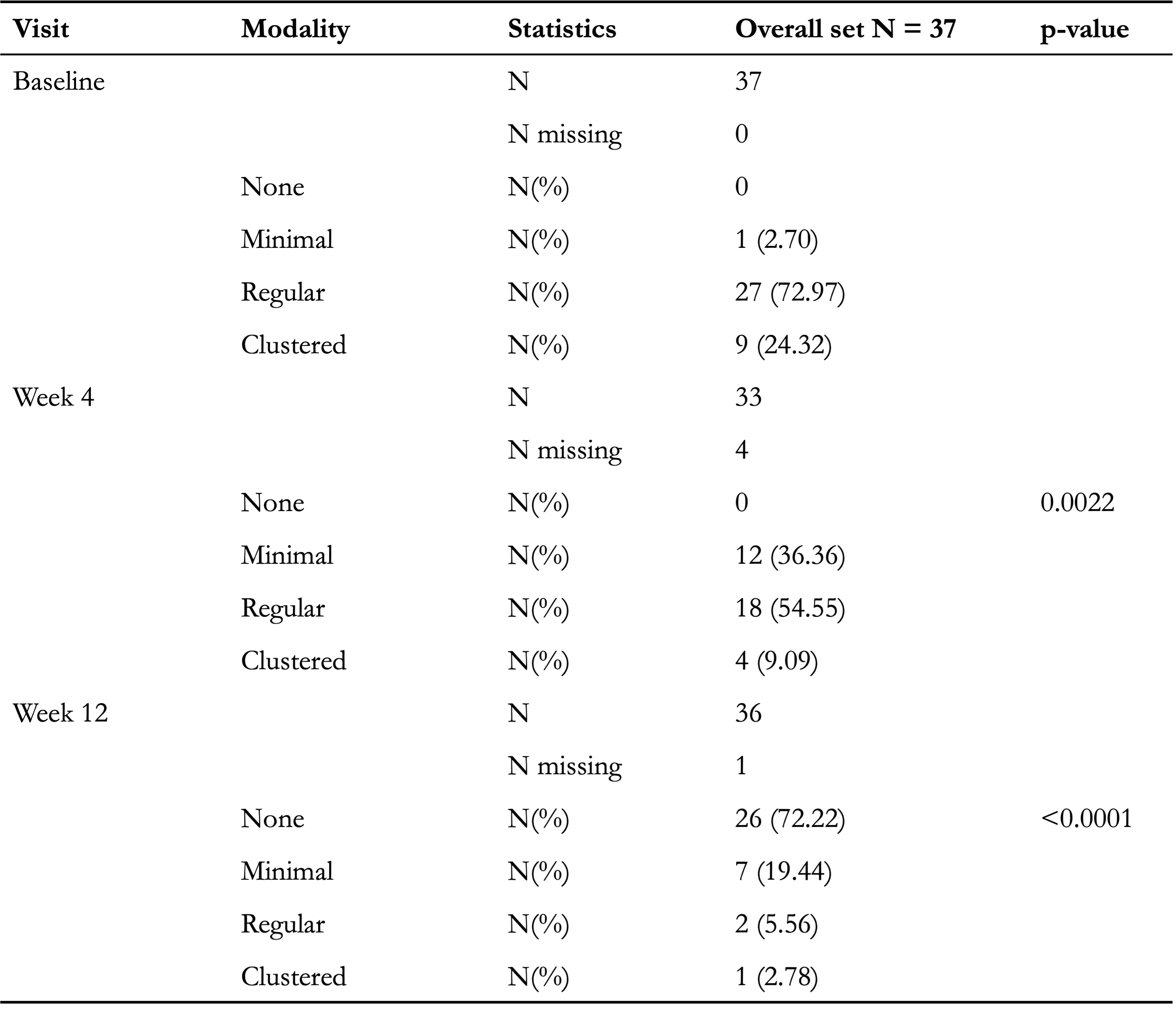

Supplement: Supplementary file 1 [file clinpract-16-00046-s001.zip › Supplementary/TableS2.png]

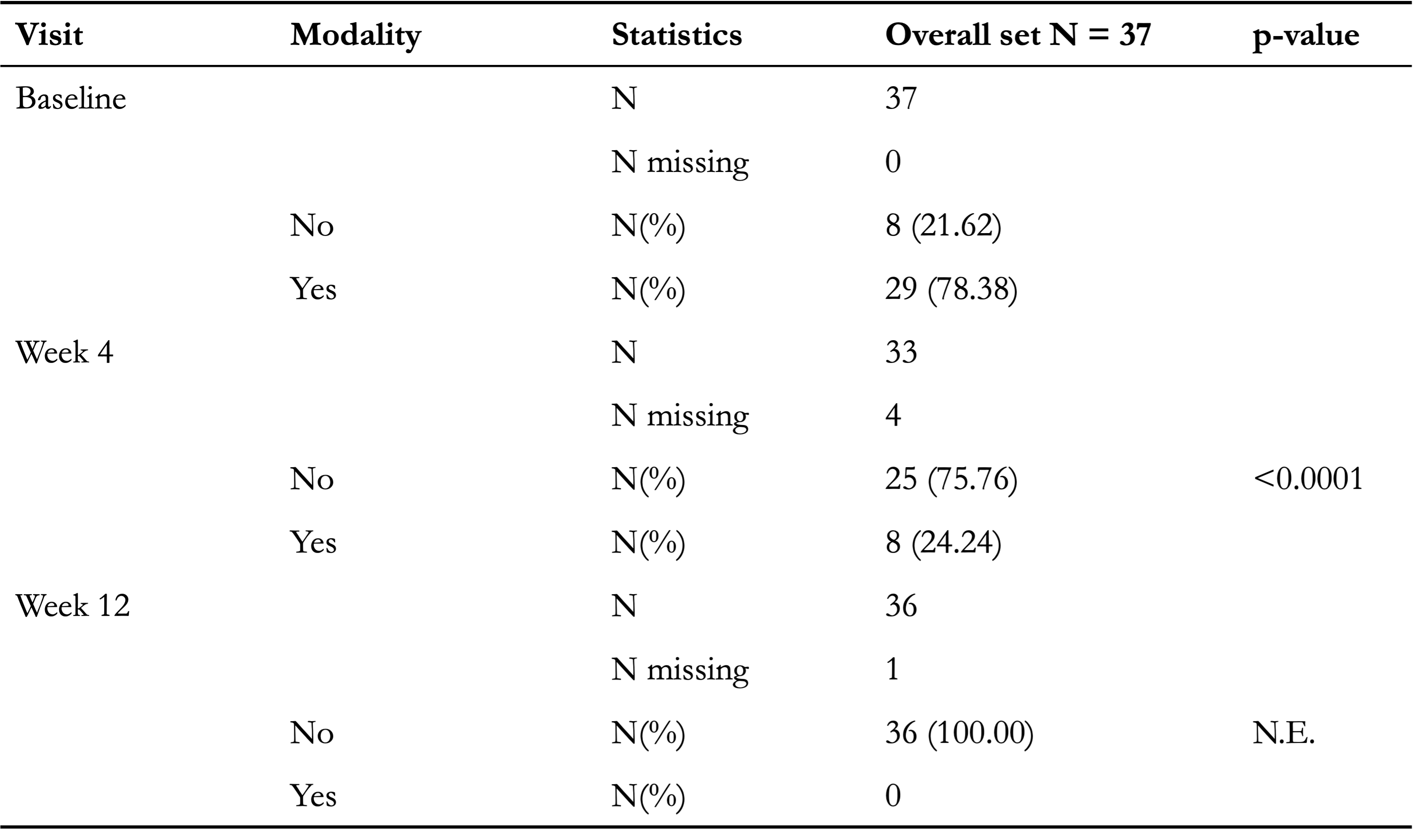

Supplement: Supplementary file 1 [file clinpract-16-00046-s001.zip › Supplementary/TableS3.png]

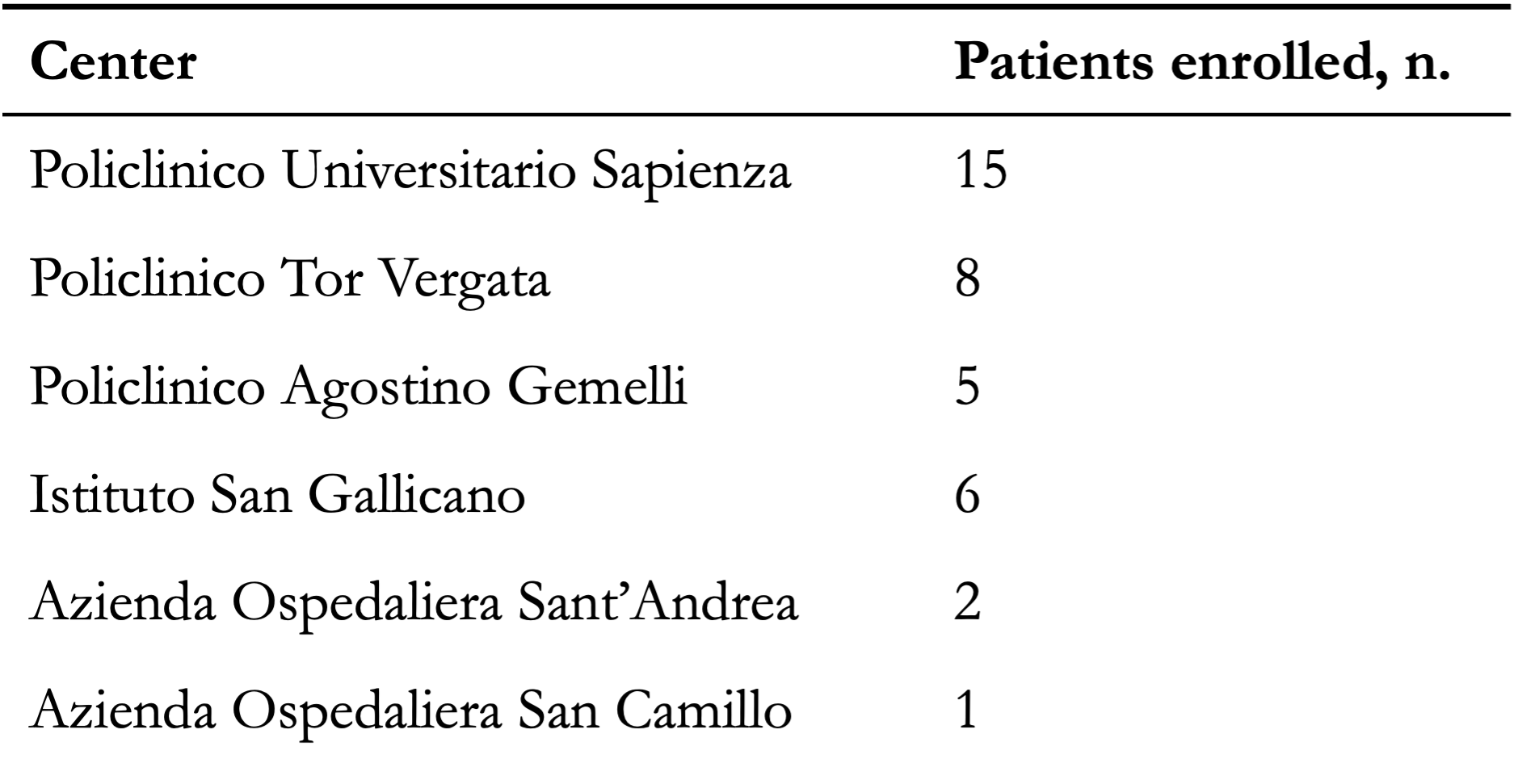

Supplement: Supplementary file 1 [file clinpract-16-00046-s001.zip › Supplementary/TableS1.png]

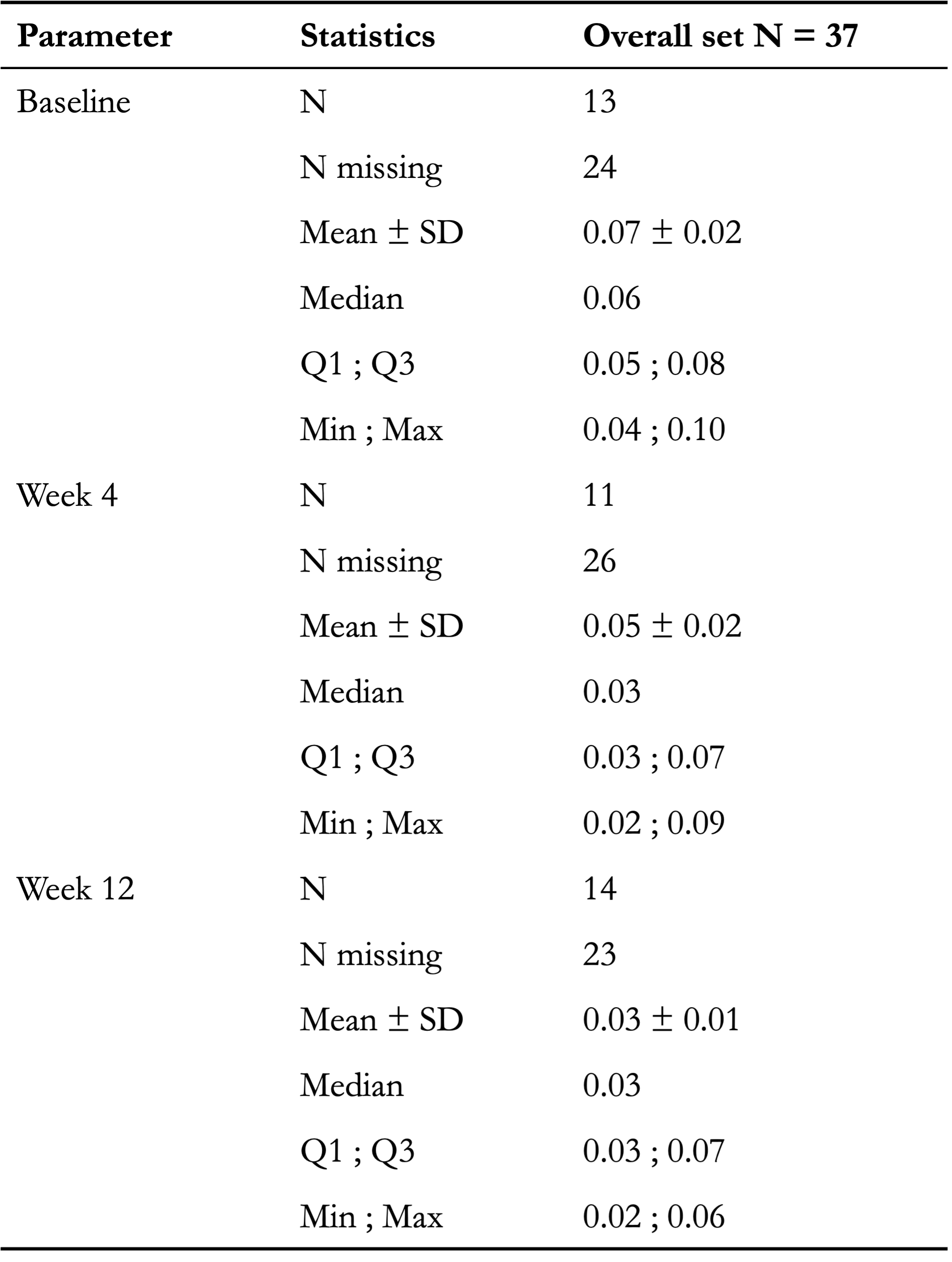

Supplement: Supplementary file 1 [file clinpract-16-00046-s001.zip › Supplementary/TableS10.png]

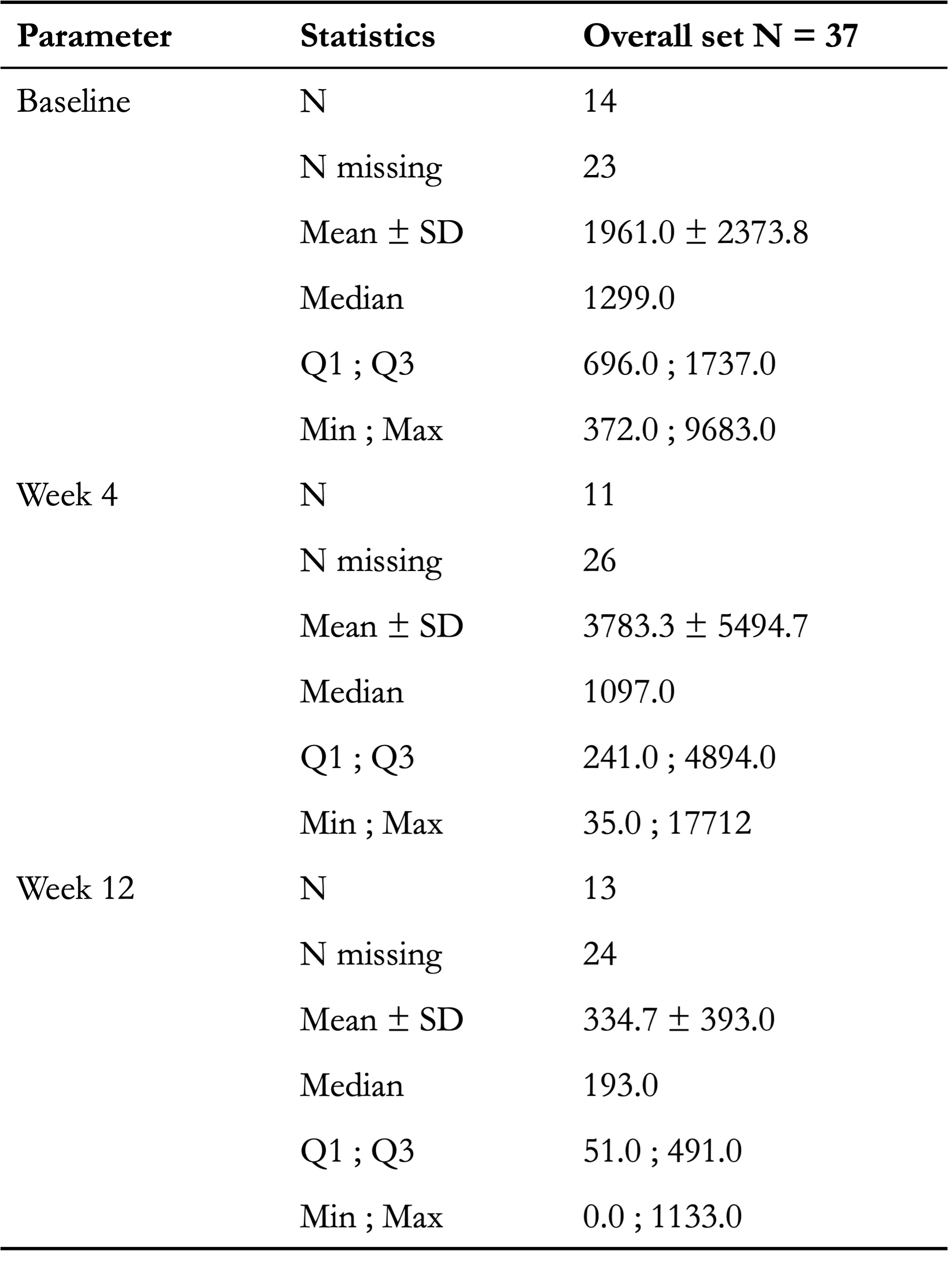

Supplement: Supplementary file 1 [file clinpract-16-00046-s001.zip › Supplementary/TableS11.png]

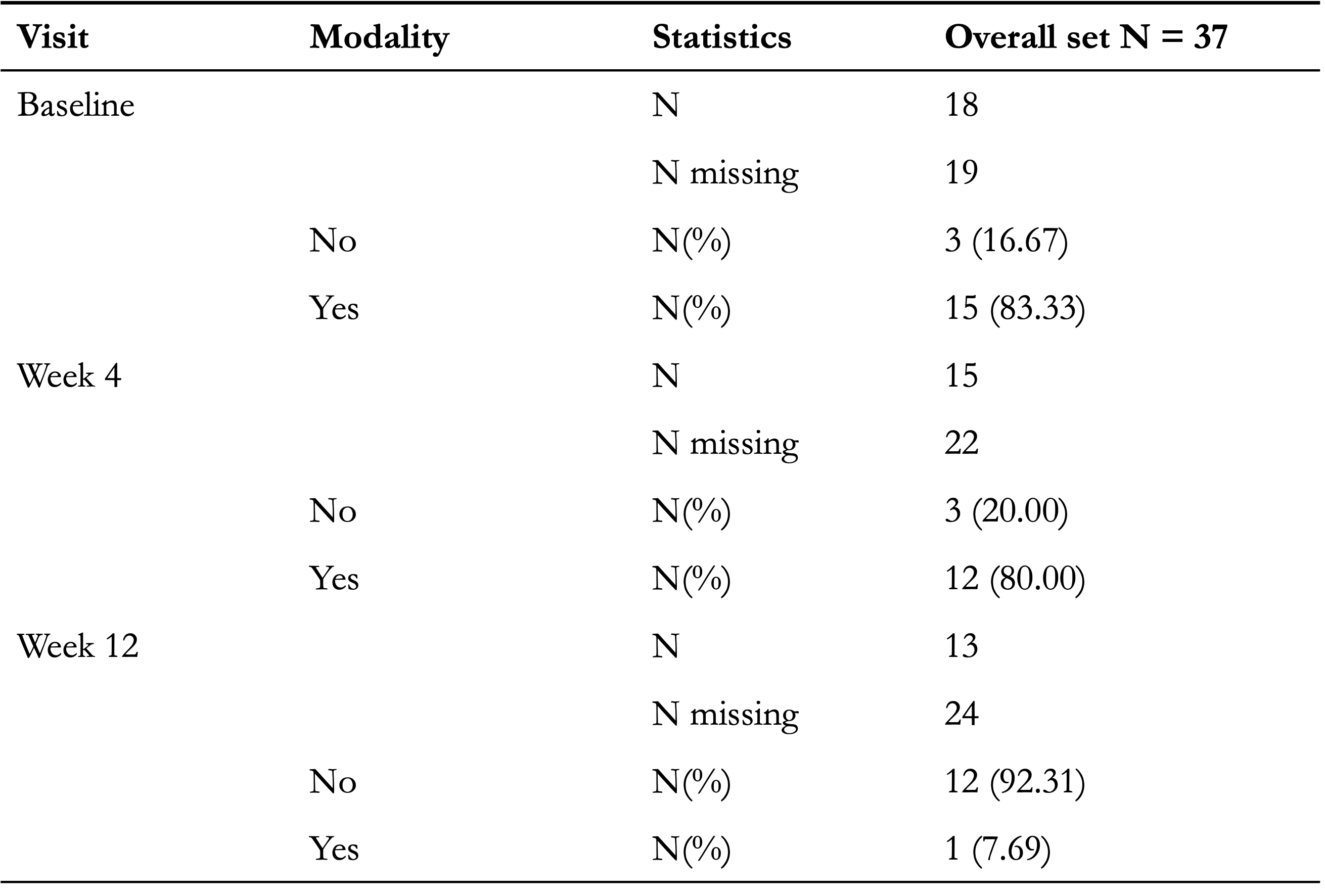

Supplement: Supplementary file 1 [file clinpract-16-00046-s001.zip › Supplementary/TableS8.png]

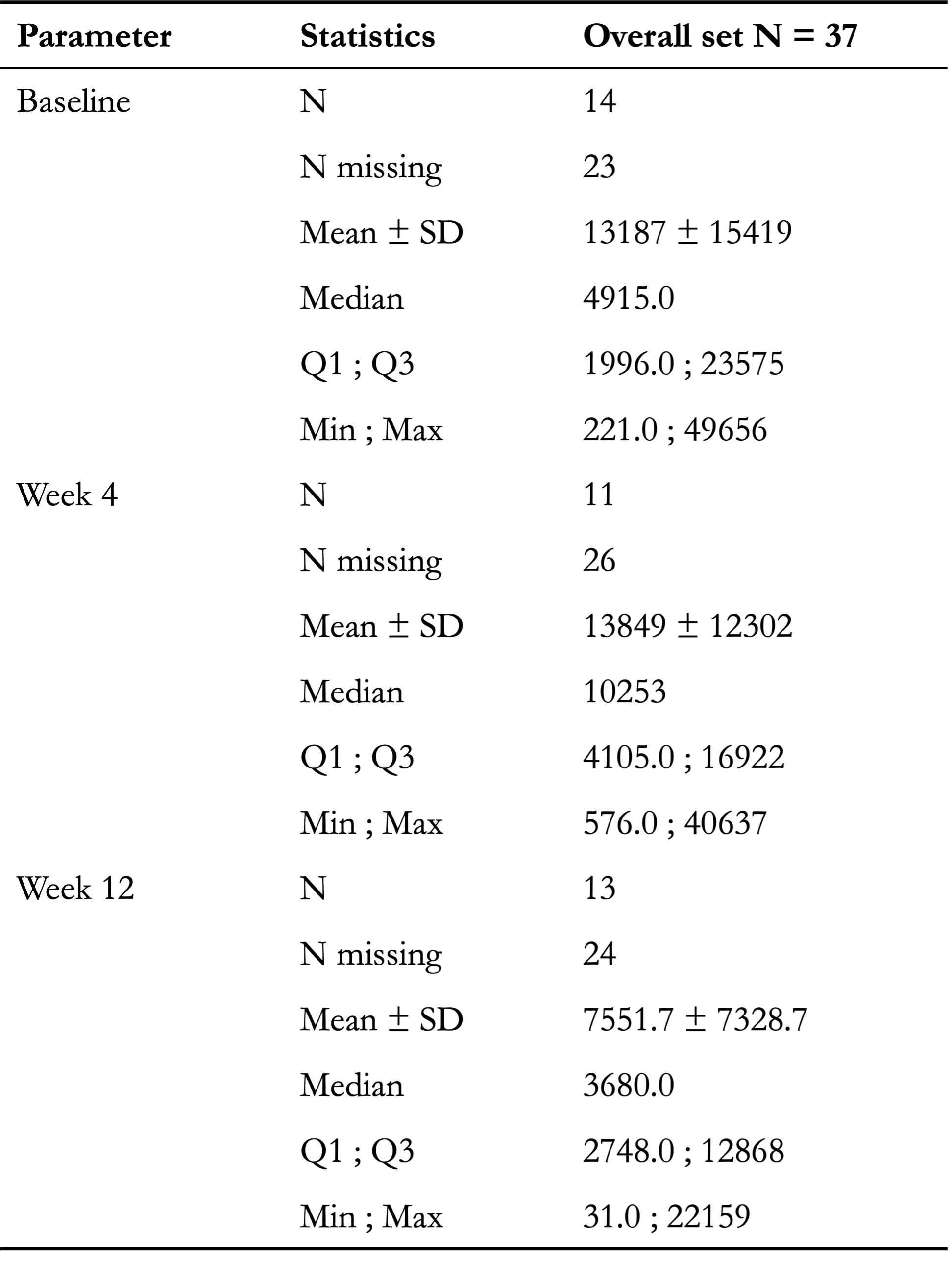

Supplement: Supplementary file 1 [file clinpract-16-00046-s001.zip › Supplementary/TableS13.png]

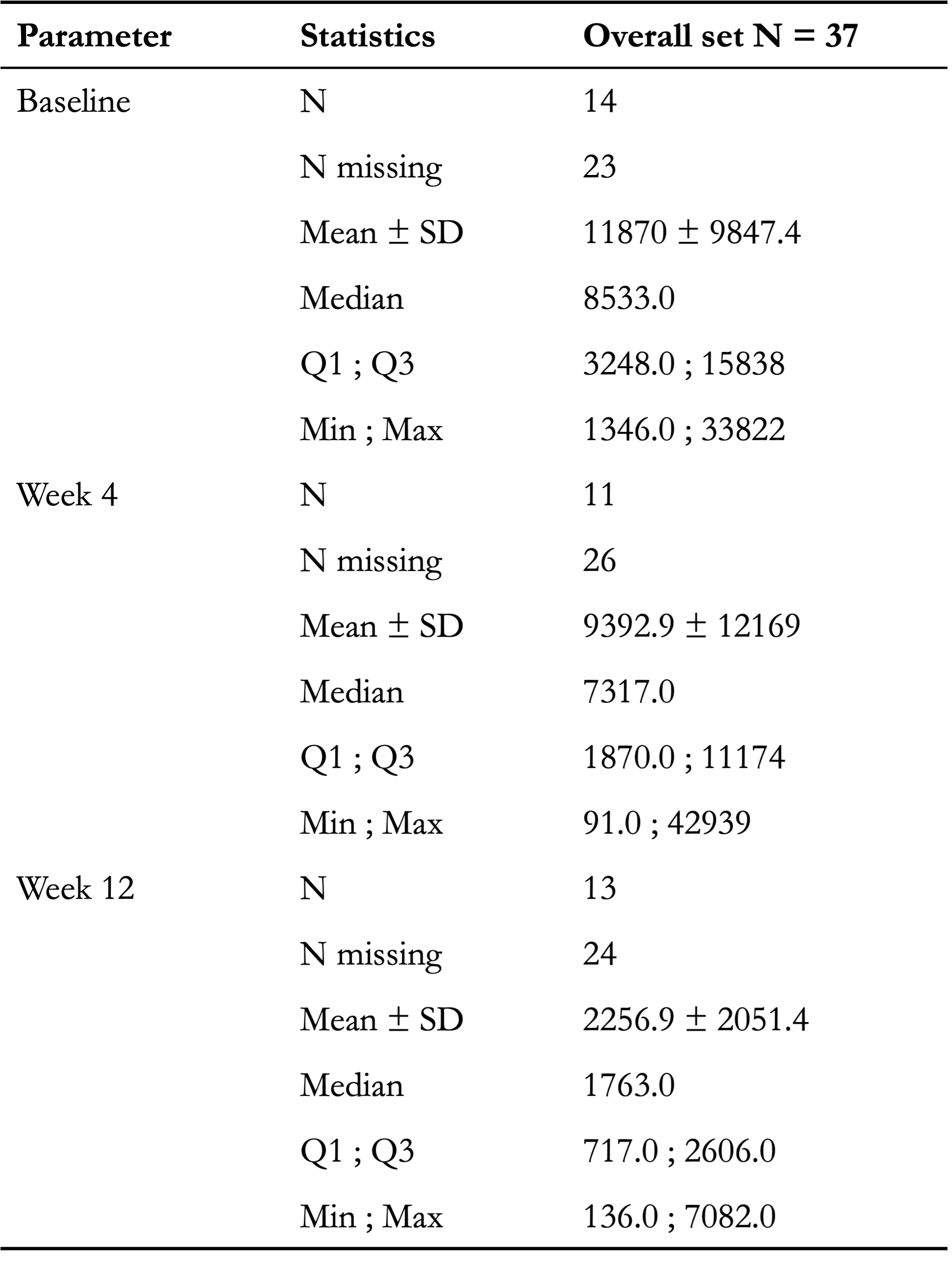

Supplement: Supplementary file 1 [file clinpract-16-00046-s001.zip › Supplementary/TableS12.png]

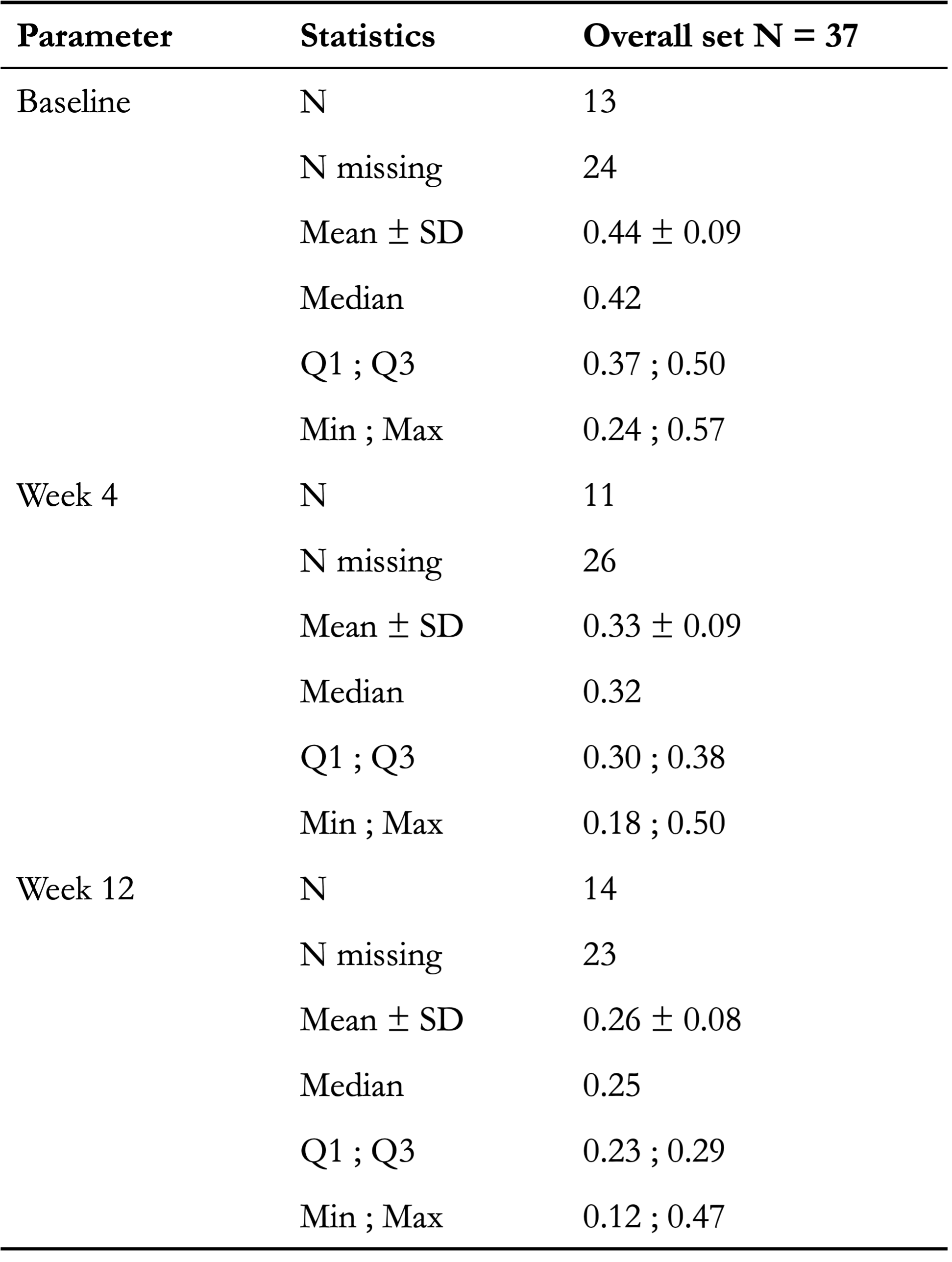

Supplement: Supplementary file 1 [file clinpract-16-00046-s001.zip › Supplementary/TableS9.png]

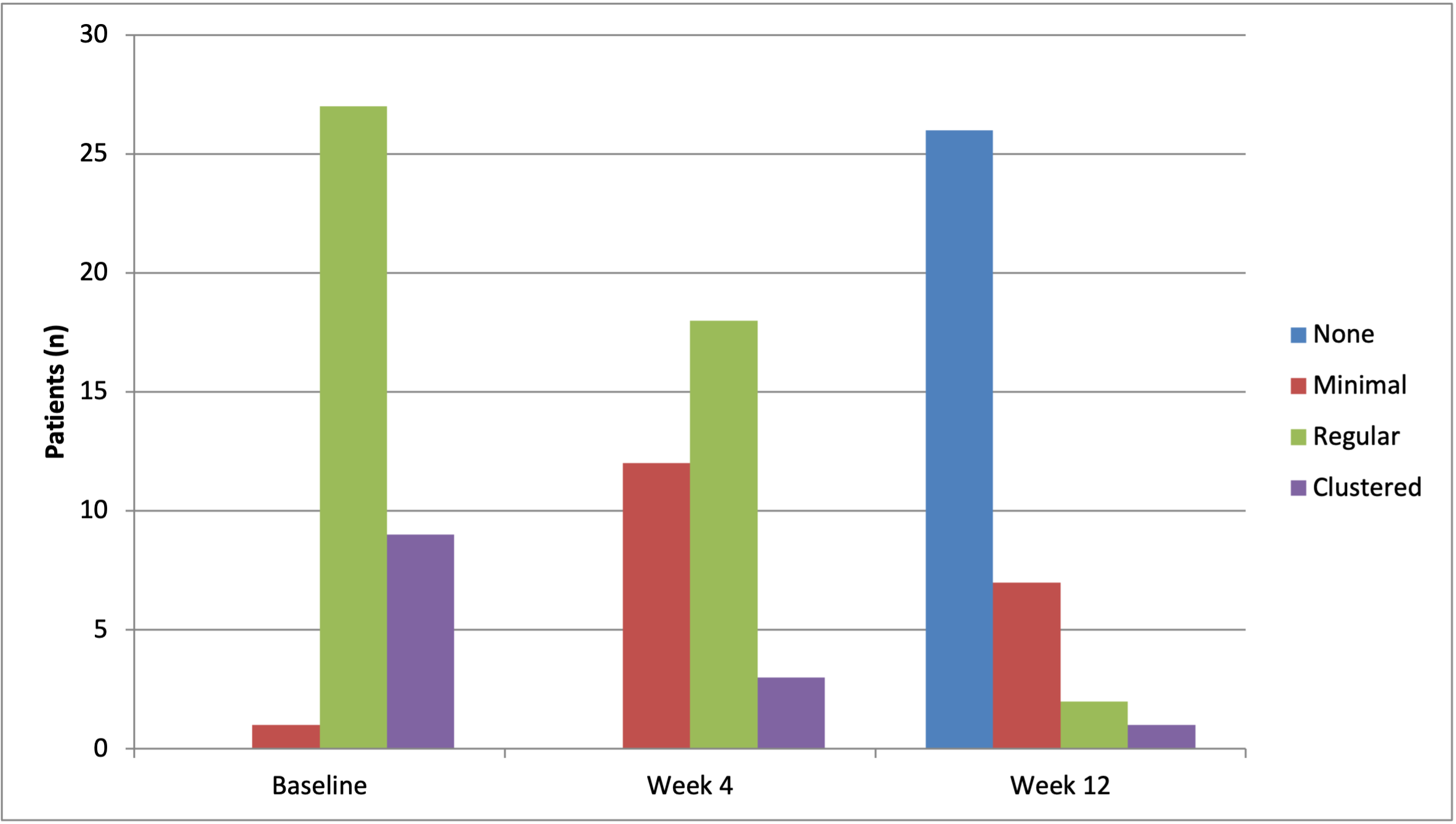

Supplement: Supplementary file 1 [file clinpract-16-00046-s001.zip › Supplementary/FigureS1.png]

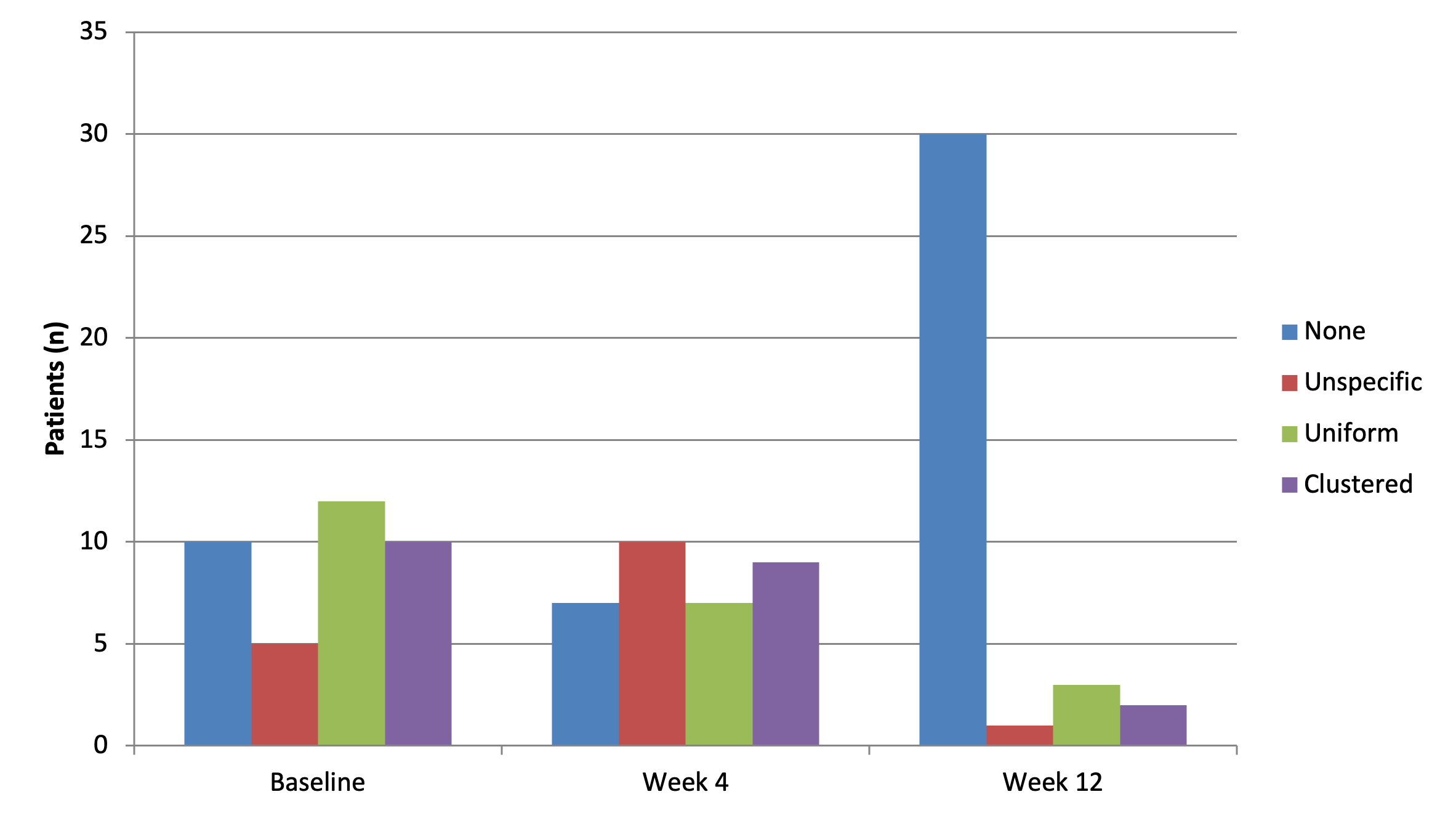

Supplement: Supplementary file 1 [file clinpract-16-00046-s001.zip › Supplementary/FigureS3.png]

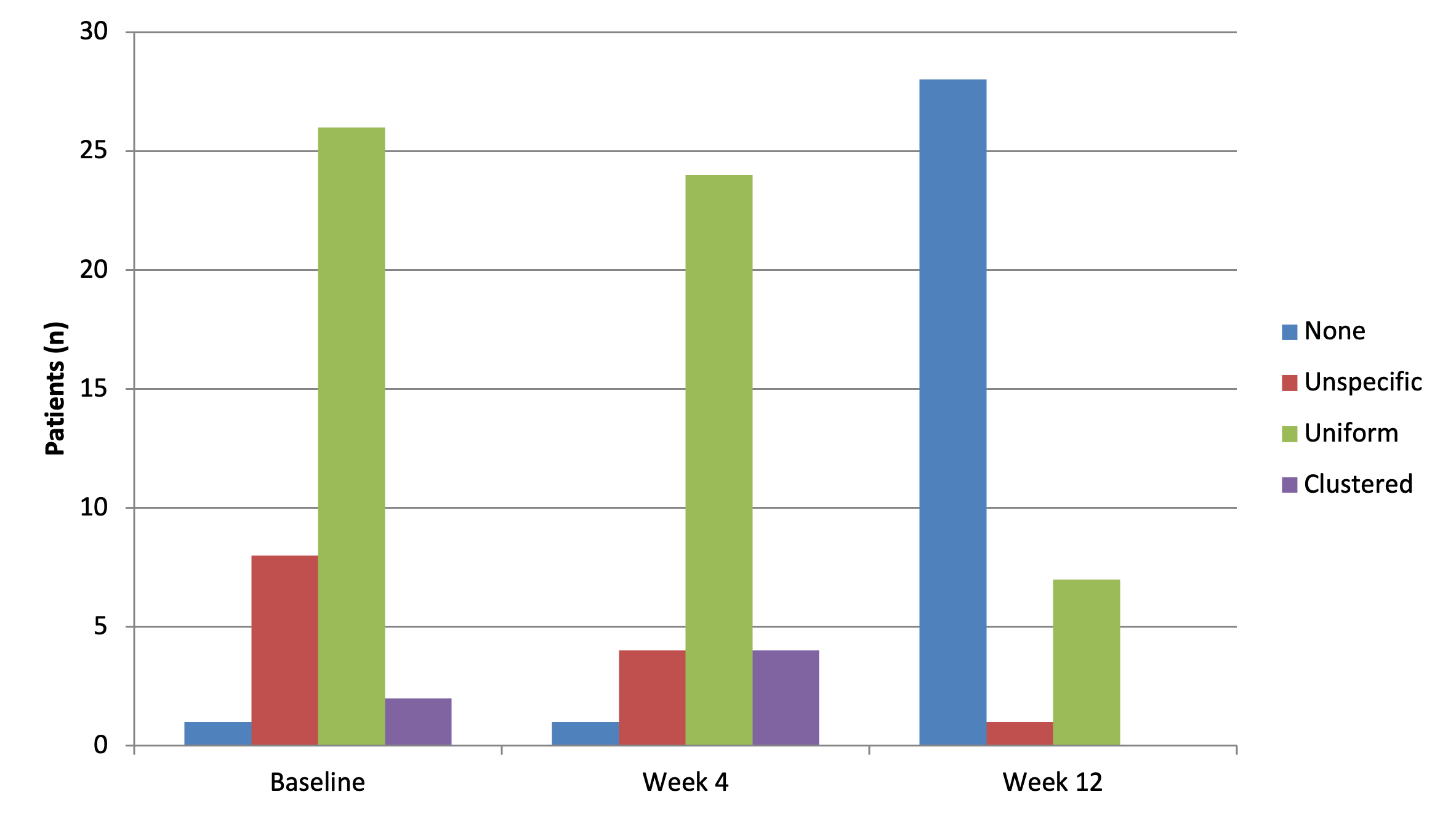

Supplement: Supplementary file 1 [file clinpract-16-00046-s001.zip › Supplementary/FigureS2.png]

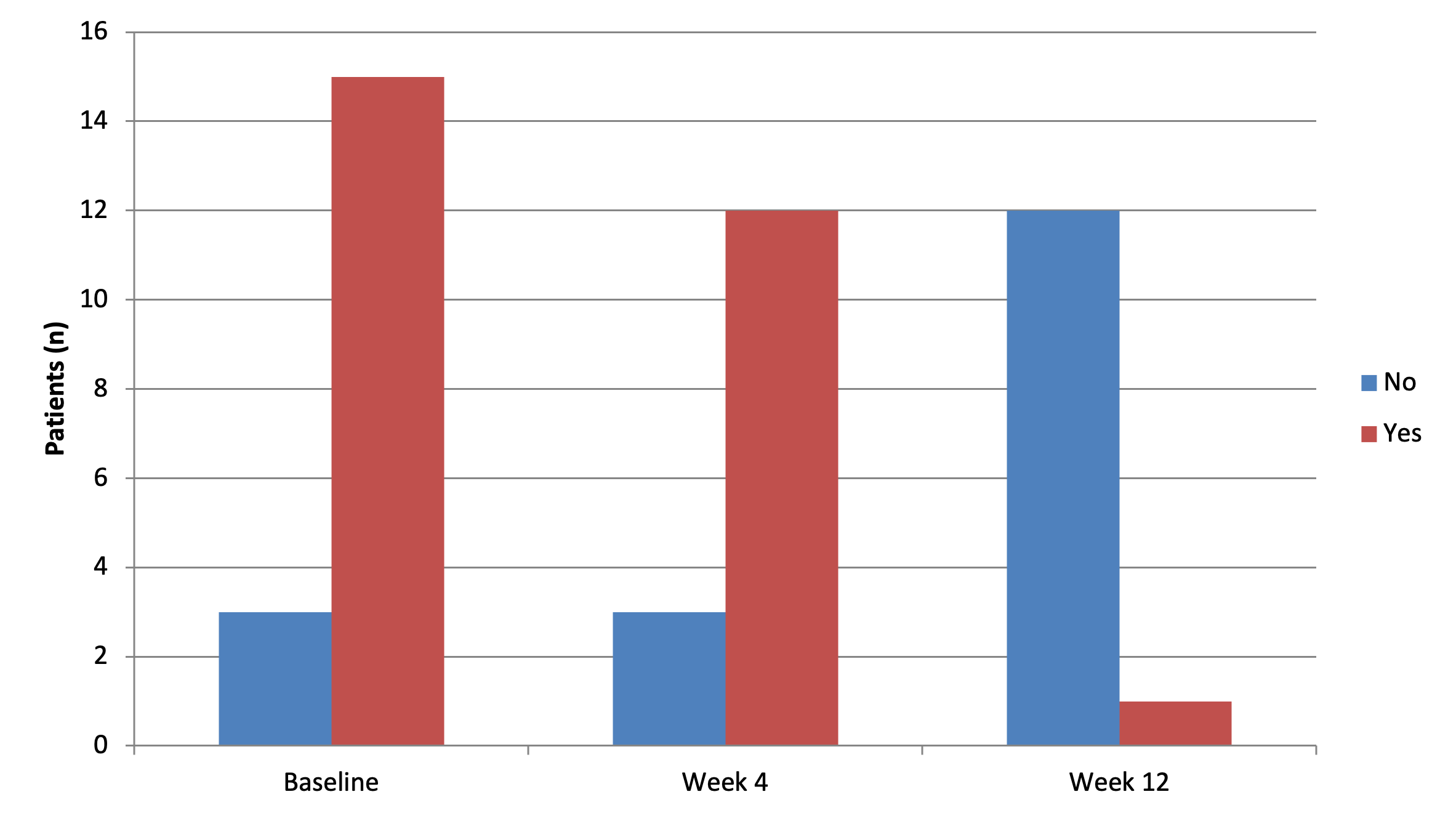

Supplement: Supplementary file 1 [file clinpract-16-00046-s001.zip › Supplementary/FigureS6.png]

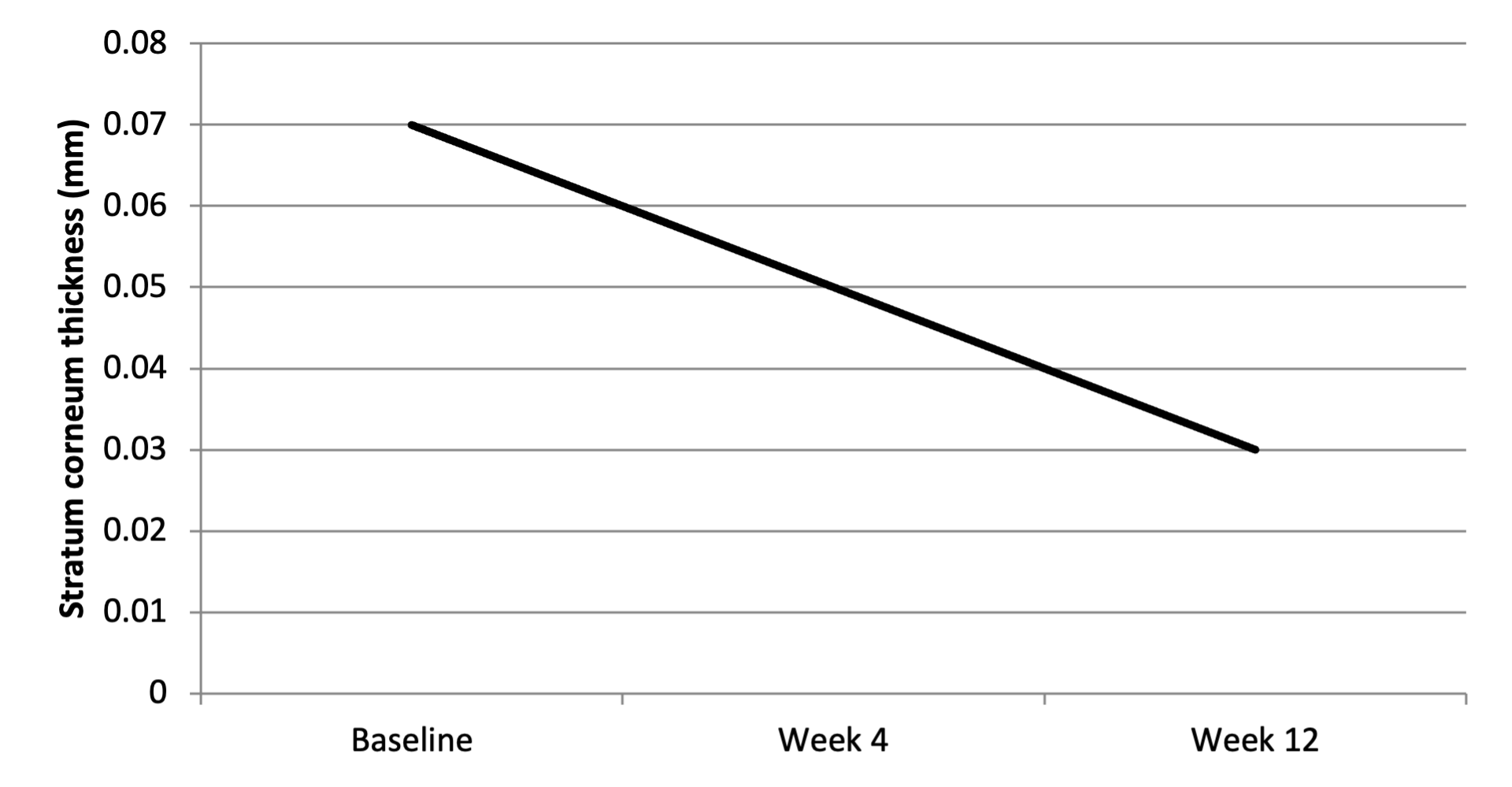

Supplement: Supplementary file 1 [file clinpract-16-00046-s001.zip › Supplementary/FigureS7.png]

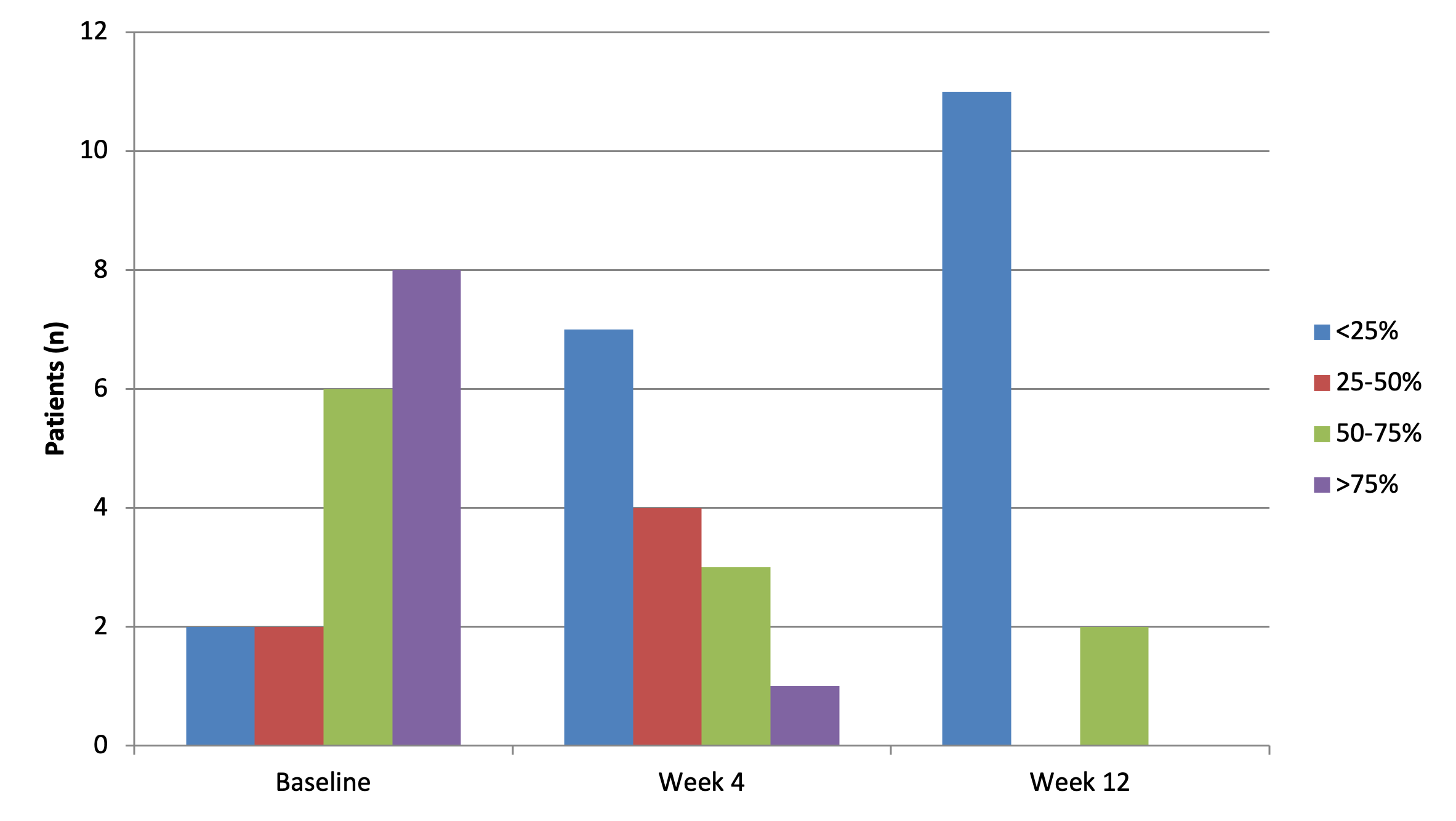

Supplement: Supplementary file 1 [file clinpract-16-00046-s001.zip › Supplementary/FigureS5.png]

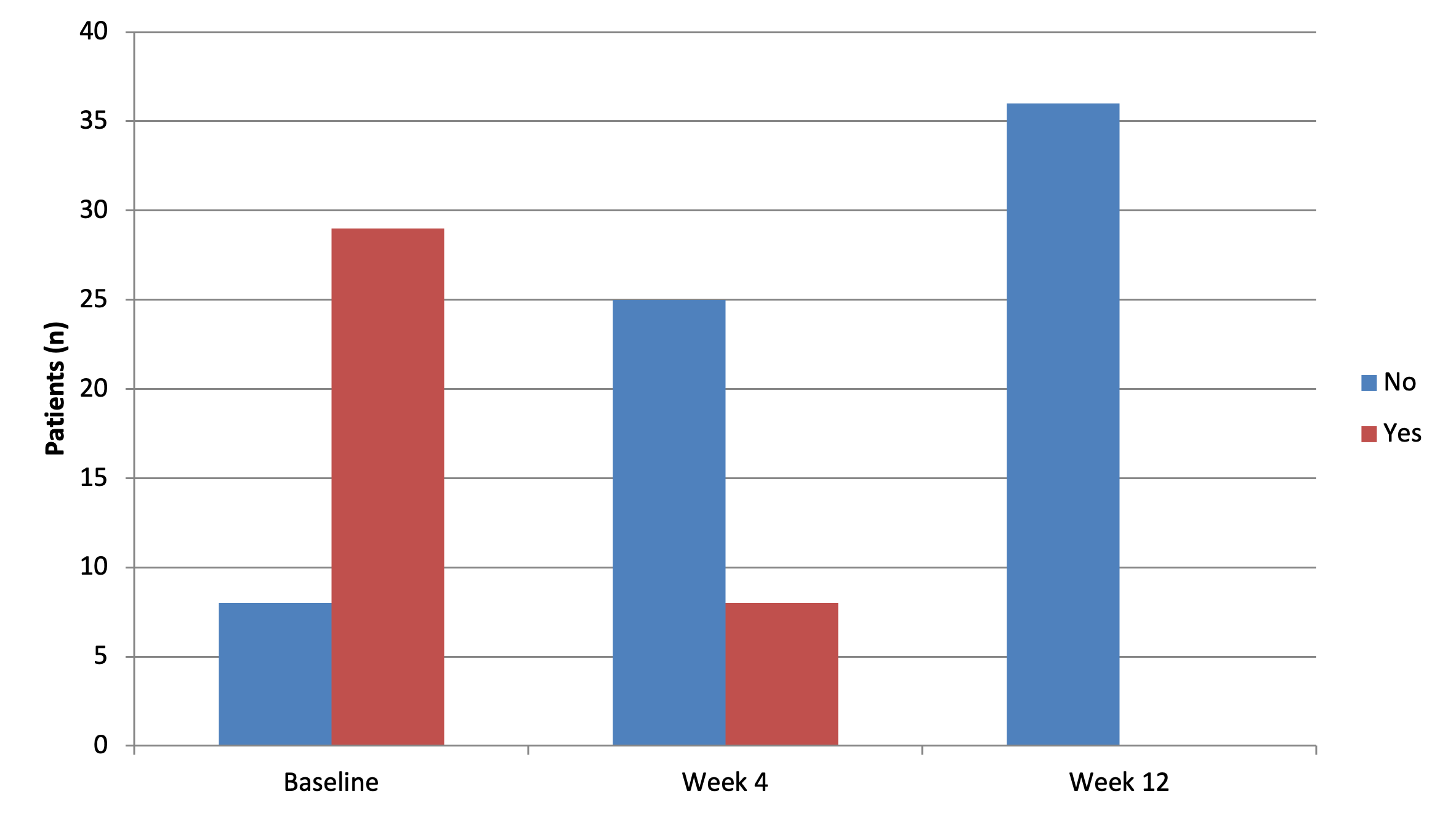

Supplement: Supplementary file 1 [file clinpract-16-00046-s001.zip › Supplementary/FigureS4.png]
